# Investigative on the Molecular Mechanism of Licorice Flavonoids Anti-Melanoma by Network Pharmacology, 3D/2D-QSAR, Molecular Docking, and Molecular Dynamics Simulation

**DOI:** 10.3389/fchem.2022.843970

**Published:** 2022-03-02

**Authors:** Yi Hu, Yufan Wu, CuiPing Jiang, Zhuxian Wang, Chunyan Shen, Zhaoming Zhu, Hui Li, Quanfu Zeng, Yaqi Xue, Yuan Wang, Li Liu, Yankui Yi, Hongxia Zhu, Qiang Liu

**Affiliations:** ^1^ School of Traditional Chinese Medicine, Southern Medical University, Guangzhou, China; ^2^ Department of Traditional Chinese Medicine, Guangzhou Red Cross Hospital, Jinan University, Guangzhou, China; ^3^ Integrated Hospital of Traditional Chinese Medicine, Southern Medical University, Guangzhou, China

**Keywords:** licorice flavonoids, melanoma, 3D-QSAR, molecular docking, MD simulation

## Abstract

Licorice flavonoids (LCFs) are natural flavonoids isolated from *Glycyrrhiza* which are known to have anti-melanoma activities *in vitro*. However, the molecular mechanism of LCF anti-melanoma has not been fully understood. In this study, network pharmacology, 3D/2D-QSAR, molecular docking, and molecular dynamics (MD) simulation were used to explore the molecular mechanism of LCF anti-melanoma. First of all, we screened the key active components and targets of LCF anti-melanoma by network pharmacology. Then, the logIC_50_ values of the top 20 compounds were predicted by the 2D-QSAR pharmacophore model, and seven highly active compounds were screened successfully. An optimal 3D-QSAR pharmacophore model for predicting the activity of LCF compounds was established by the HipHop method. The effectiveness of the 3D-QSAR pharmacophore was verified by a training set of compounds with known activity, and the possible decisive therapeutic effect of the potency group was inferred. Finally, molecular docking and MD simulation were used to verify the effective pharmacophore. In conclusion, this study established the structure–activity relationship of LCF and provided theoretical guidance for the research of LCF anti-melanoma.

**GRAPHICAL ABSTRACT:**
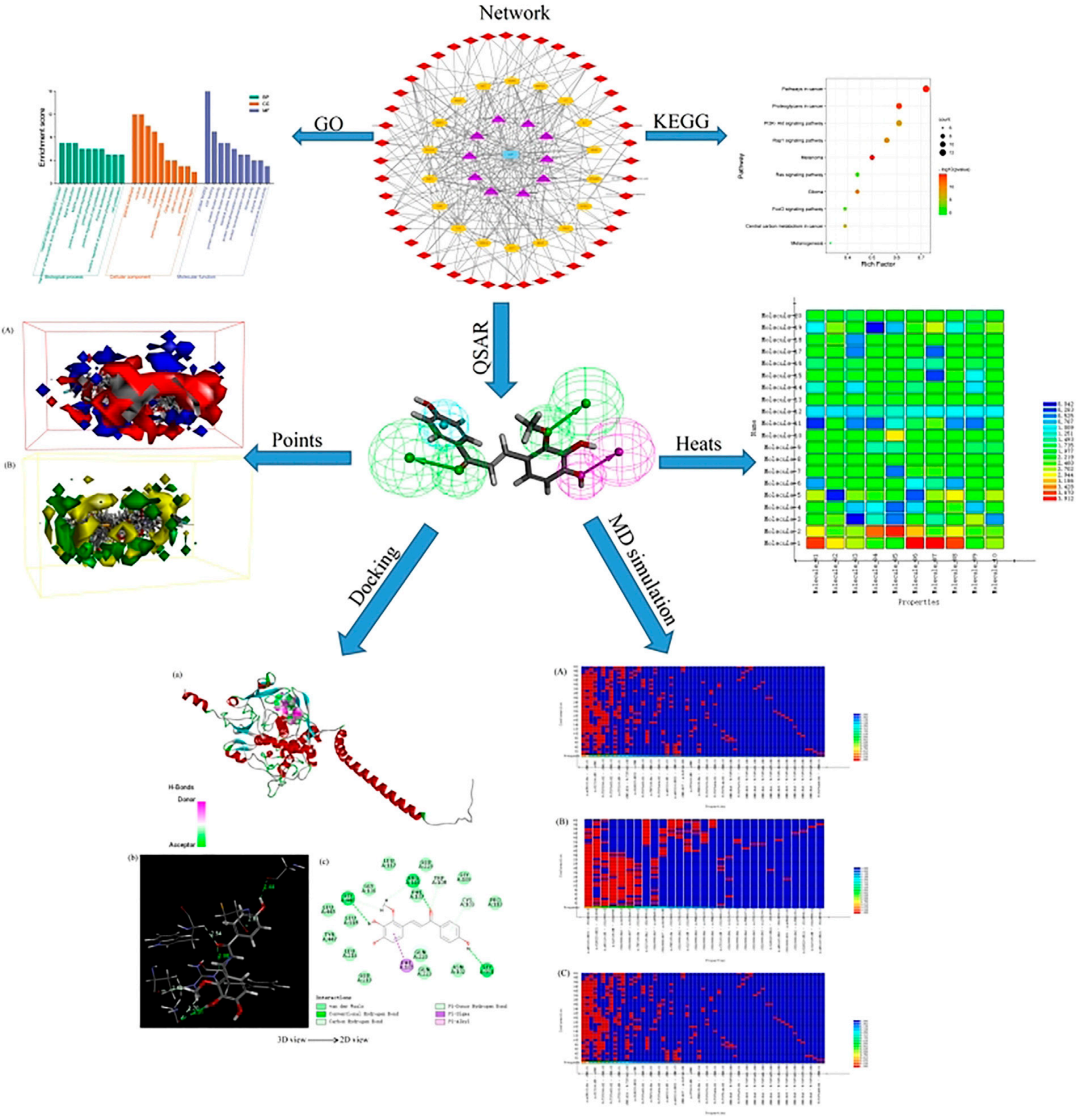


## 1 Introduction

Melanoma is a common skin cancer with a high annual mortality rate, accounting for 80% of all skin cancer deaths, and is considered a serious health problem (Rastrelli et al., 2014). At present, surgical resection of melanoma is the most common treatment, but most patients with advanced melanoma are ineffective and prone to recurrence (Chen et al., 2019). In addition, the use of multidisciplinary methods such as chemotherapy, radiotherapy, and immunotherapy of melanoma is also a common treatment, but it will have an irreversible impact on skin, tissue, and organs (Liu et al., 2013; Lang et al., 2019). Therefore, more and more researchers have paid attention to finding an effective alternative therapy for melanoma (Naidoo et al., 2018; Zhang et al., 2018; Song et al., 2021).

In recent years, natural products have been widely used in medicine and pharmacology because of their potential chemotherapeutic activity (Kang et al., 2017). As an alternative therapy in modern medicine, natural products have been shown to have better antitumor activity with fewer side effects (Cho et al., 2015). LCF is a kind of natural active ingredient extracted from *Glycyrrhiza* and has a variety of biological activities, such as antioxidant, hepatotoxicity, anti-inflammatory, anti-ulcer, anti-allergy, antivirus, antitumor, and other biological activities (Uto et al., 2019). For example, among LCFs, licochalcone B reduces inflammation, migration, angiogenesis, and tumorigenesis and induces cell cycle arrest and apoptosis of various cancer cells *in vitro* and *in vivo* (Yuan et al., 2014; Oh et al., 2016). Isoliquiritigenin can inhibit cell proliferation and induce cell apoptosis by reducing hypoxia and glycolysis in B16F10 mouse melanoma cells (Wang et al., 2016). Although these studies have proved that LCFs have an antitumor biological function, there are few reports available on the molecular mechanism of LCF anti-melanoma.

Network pharmacology is a new subject derived from system biology, which combines computer biology with network analysis (Lv et al., 2020; Jiao et al., 2021). It can explain the pharmacological mechanism of drugs on complex diseases through multicomponent, multi-target, and multi-approach (Xia and Tang, 2021). Quantitative structure–activity relationship (QSAR) is the use of mathematical statistics to study and reveal the quantitative laws of change between the activity of a compound and its molecular structure or physicochemical characteristics, thereby allowing these “laws” to be used to assess new chemical entities (Wang et al., 2015; Alves et al., 2016; Yan et al., 2020). Therefore, if the bioactivity data of a series of structural analogs can be collected, the QSAR method can be used to predict the related activity of unknown compounds (Muratov et al., 2020; Yadav et al., 2020). For example, Tawassl et al. established a 2D-QSAR model of a pyrazole kinase inhibitor (EGFR) containing a thiourea skeleton using the QSAR method and successfully predicted its bioactivity as an EGFR kinase inhibitor using the 2D-QSAR model (Hajalsiddig et al., 2020). Gao et al. used known tyrosinase inhibitors to generate a 3D-QSAR model and successfully screened out tyrosinase inhibitors with high activity (Gao, 2018). Molecular docking is a method to predict the binding posture and affinity between the receptor protein and ligand through the interaction between the ligand and receptor, which can be used to explain the mechanism of action between drug targets (Ren et al., 2019; Avdovic et al., 2021). Molecular dynamics (MD) is one of the most commonly used methods in molecular simulation. This method is a key theoretical method to evaluate the stability and flexibility of molecules by dynamically describing the motion of molecules based on the molecular force field (Hildebrand et al., 2019; Avdović et al., 2022).

In this study, we screened the key active components and targets of LCF anti-melanoma by network pharmacology. Then, the logIC_50_ of tyrosinase was successfully predicted by the 2D-QSAR pharmacophore model, and the optimal 3D-QSAR pharmacophore model for predicting the activity of LCF compounds was constructed by the HipHop method. Finally, the molecular mechanism of LCF anti-melanoma was revealed by molecular docking and MD simulation.

## 2 Materials and Methods

### 2.1 Network Pharmacology

In order to clarify the complex relationship between LCF and melanoma-related targets, network pharmacological methods were used to analyze the network. The UHPLC–Orbitrap-MS method was used to detect the relevant chemical components of LCF (Supplementary Table S1), and the compound was identified, and its structure was downloaded using the PubChem database (Supplementary Table S2), which was saved in the SDF format. The Swiss database was used to predict the target of each compound. Search for “melanoma” in the GeneCards database to collect relevant targets (relevance score >15). Draw a Venn diagram for the predicted targets of active ingredients and disease-related targets, take the intersection targets (Supplementary Figure S1; Supplementary Table S3), and get the potential target of LCF anti-melanoma. Then, enter the potential targets for melanoma treatment into the STRING database, and set the target as “Homo” to construct a protein–protein interaction (PPI) diagram of potential targets for melanoma treatment (Supplementary Figure S2). Potential target pathway enrichment was generated through the DAVID 6.8 database (Wang et al., 2021). Finally, Cytoscape v3.8.2 software (https://cytoscape.org/download.html) was used to construct a “C-T-P" network of 44 compounds, 18 targets, and 10 signaling pathways closely related to melanoma to elucidate the effective mechanism of LCF anti-melanoma. Among them, the “C-T-P” network diagram involves 73 nodes and 257 edges, and these “nodes” are used to represent compounds, targets, and paths. Associations between nodes are represented by an edge that analyzes the degree of association between nodes based on the degree value (Wang et al., 2020).

### 2.2 Construction of the QSAR Pharmacophore Model

**FIGURE 1:**
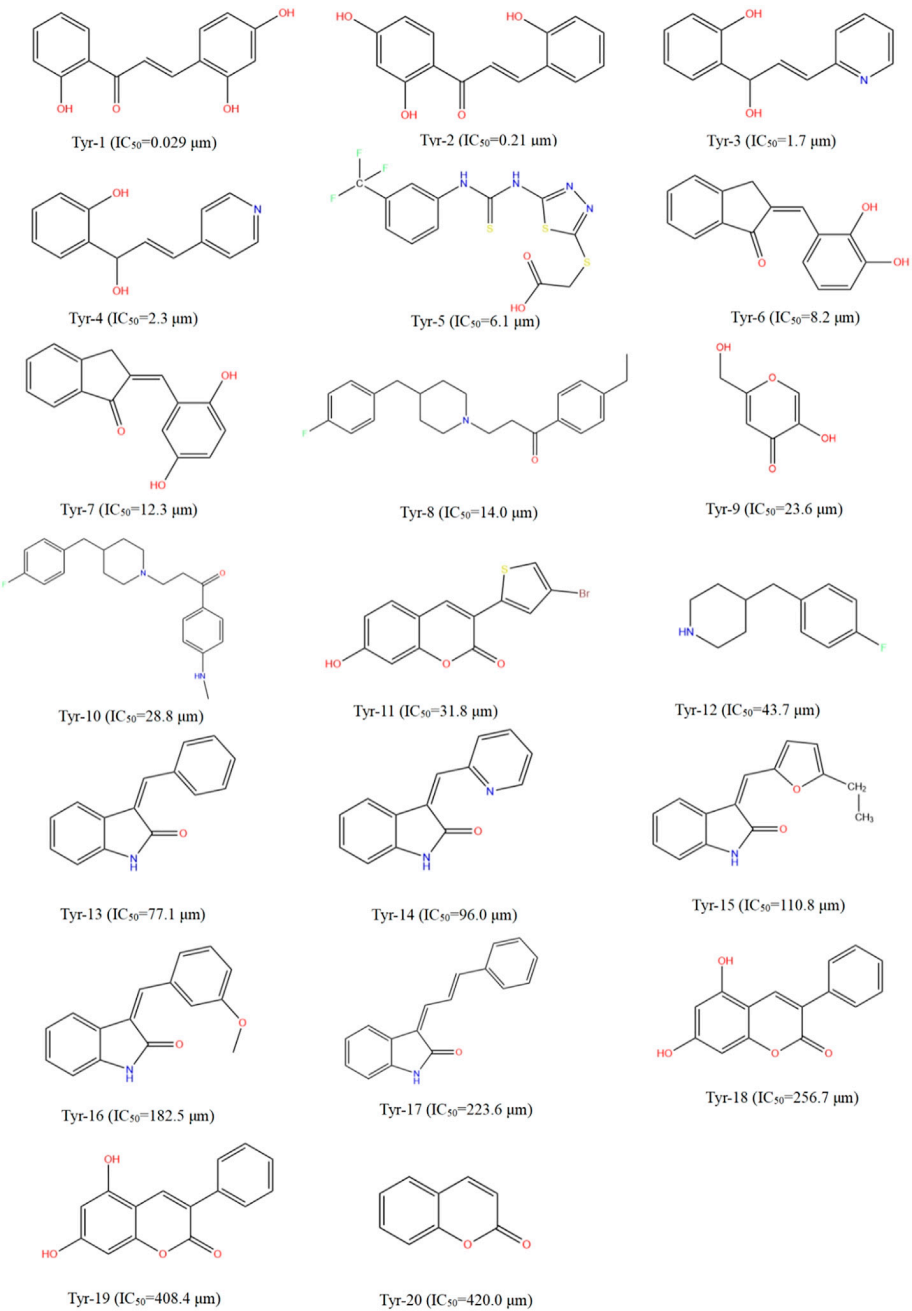
Structural formulae and corresponding IC_50_ values of 20 training set compounds.

**FIGURE 2:**
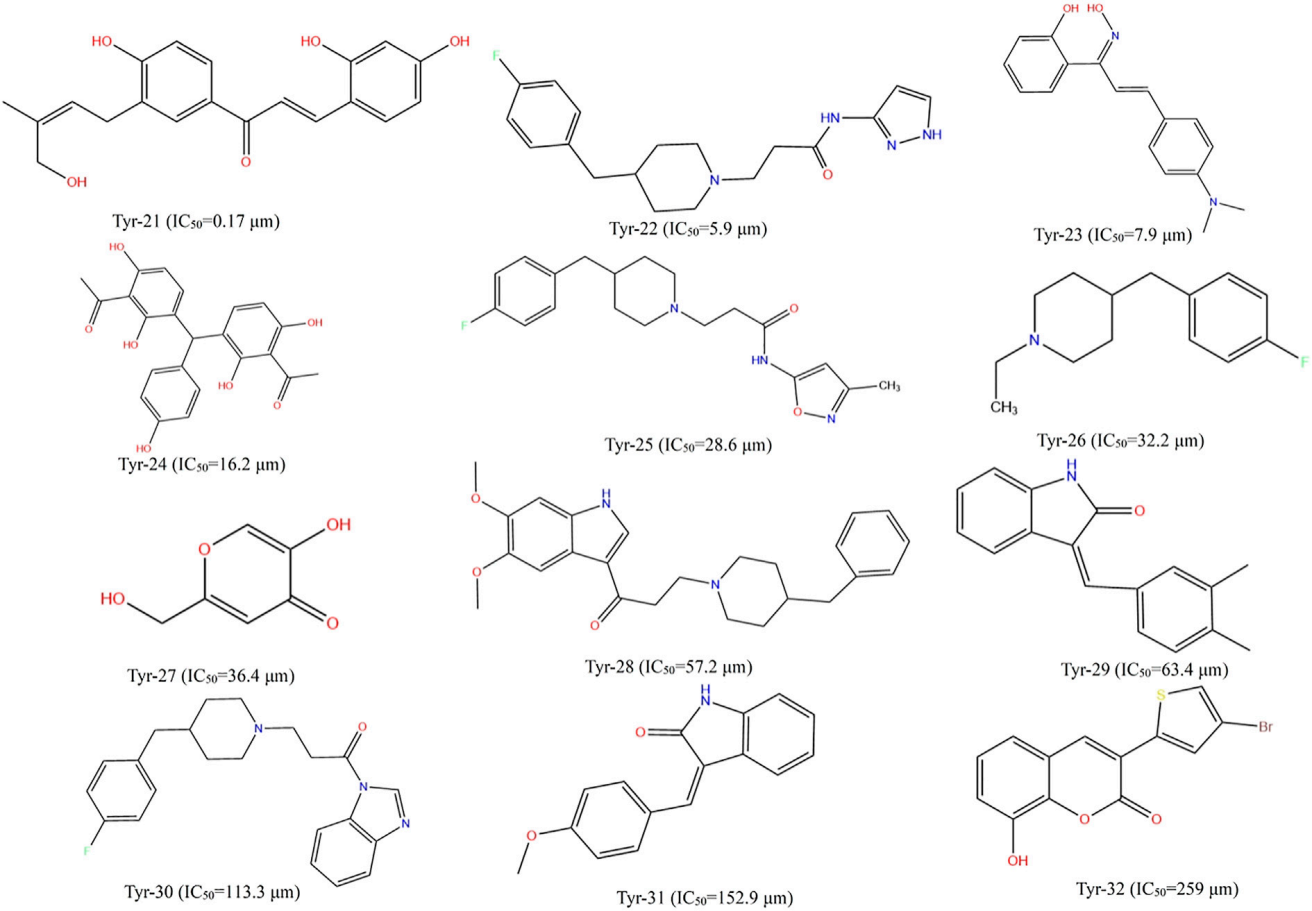
Structural formulae and corresponding IC_50_ values of 12 test set compounds.

Different datasets of 32 experimentally identified tyrosinase inhibitors were obtained from the published literature (Gao, 2018; Zolghadri et al., 2019) (Figures 1, 2). The molecules were drawn by the ChemDraw module in ChemOffice, and the energy was minimized by the Minimization module in Discovery Studio software (Discovery Studio 2019; BIOVIA; San Diego, USA). The obtained conformations were used for subsequent analysis. All molecular modeling calculations were performed by Discovery Studio software. Compounds of 20 tyrosinase inhibitors were used to construct training sets for the formation of QSAR pharmacophores. To ensure the accuracy of the model, the selected tyrosinase inhibitor activity values span four quantity sets (0.029–420 μm), and 12 other compounds of tyrosinase inhibitors were selected to construct a test set (0.17–259 μm) to generate QSAR pharmacophore. The QSAR method was based on multiple linear regression (MLR), partial least squares (PLS), and other statistical methods to reveal the quantitative change law between the activity of compounds and their molecular structure or physicochemical characteristics. Therefore, in the construction of the QSAR model, the biological activity represented by the logIC_50_ value was the dependent variable, and its corresponding physical and chemical properties were the independent variables (Lagunin et al., 2018).

#### 2.2.1 Construction of the 2D-QSAR Pharmacophore Model

This section uses Discovery Studio software to build the 2D-QSAR model. A computational molecular property module was used to calculate the 2D molecular properties of the training set and the test set (Taxak and Bharatam, 2013). Multiple linear regression (MLR) and partial least squares (PLS) were used to construct the structure–activity relationship model (Hajalsiddig et al., 2020). The biological activity of a compound was defined as logIC_50_, and the physicochemical parameters used in establishing the structure–activity relationship were the 2D properties of the compound calculated in the software, such as AlogP, molecular properties (molecular weight), molecular property count (Num_Aromic Rings, Num_-H_Acceptors, Num_H_Donors, Num_Ring, and Num_Rotatable Bond), and surface area and volume (Moner _ Decononal Surface) (Imran et al., 2015).

#### 2.2.2 Construction of the 3D-QSAR Pharmacophore Model

The 3D-QSAR pharmacophore model was constructed using the HipHop method of the Common Feature Pharmacophore Generation module under the Pharmacophore item in Discovery Studio software (Kim et al., 2021). The HipHop method constructs a pharmacophore model based on the known 3D structure and bioactivity data of a series of compounds and describes the common characteristics of their bioactivity. The established pharmacophore model can be used to explore the structure of the compound and its biological activity (Wang et al., 2008). Briefly, set the principal value of a compound with IC_50_ below 1 μm to 2 and the MaxOmitFeat value to 0 (all chemical characteristics of the compound are considered when constructing the pharmacophore model, and all the characteristic elements of the established pharmacophore must be matched). Set the principal and MaxOmitFeat values of compounds higher than 1 μm for IC_50_ to 1 (the conformational space should be referred to when modeling, but the modeling result can have a characteristic element that does not match it). Feature mapping was used to identify the characteristic elements of the training set, to study the molecules, including those main characteristic elements, and then set the obtained characteristic elements as the characteristic elements of the pharmacophore effect to be considered by HypoGen, namely hydrogen bond receptor (HBA), hydrogen bond donor (HBD), hydrophobic center (H), cationic group (PI), and aromatic ring center (R) were five items as possible pharmacophore characteristic elements (Fan et al., 2018). The range of each pharmacodynamic element was 0–5. The upper limit of 255 conformations for each compound was set, and only conformations with an energy difference of 10 kcal mol^−1^ from the lowest conformations were preserved. After calculation, only 10 pharmacophore models with the highest scores were retained (Jiang et al., 2016). The established pharmacophore model was verified by using the training set and ligand analyzer calorimeter. A good pharmacophore model should have a high matching performance to the active compounds.

### 2.3 Molecular Docking

A total of seven active molecules predicted by the pharmacophore model were selected as ligand molecules, and the Tyr with the highest score screened by network pharmacology was selected as a receptor protein, and the structure of tyrosinase (EC 1.14.18.1) protein was downloaded from PubChem. The Full Minimization module of Discovery Studio is used to minimize energy of small molecules, and the CHARMM force field is set to assign to the structure. This structure is used as the starting conformation to perform molecular docking (Ahmed et al., 2017). Molecular docking was performed using Discovery Studio software (Saxena et al., 2018). LibDockScore ≥90 indicates that the ligand and receptor affinity is strong, and the ligand binding is easier (Chen peng et al., 2021). The smaller the docking bond energy is, the more stable the complex of the ligand and protein is (Chen Weijian et al., 2021). The result with the highest score for molecular docking will be presented as the final conformation, from which the interaction energy after docking can be calculated. The associated free energy can be calculated by Eq. 1 (Pal et al., 2019).

$$\Delta {\text{G}}_{\text{Binding}}={\text{E}}_{\text{Complex}}-\left({\text{E}}_{\text{Protein}}+{\text{E}}_{\text{Ligand}}\right),$$ (1)

### 2.4 MD Simulation

MD simulations were performed using the Standard Dynamics Cascade module of Discovery Studio software for protein and ligand complexes with the highest fractions after molecular docking (El et al., 2020), to explore the stability of ligand molecules in proteins. The system was modeled using an extended simple point charge (SPC/E) water model in which the entire system was placed in a solvent chamber with a periodic boundary filled with water molecules and further stabilizes the charge using Cl^−^ and Na^+^ to keep the entire simulation system electrically neutral. Initially, the steepest descent method was used to minimize the energy of the entire system (Barcellos et al., 2019). The system was then balanced by the NVT ensemble (constant number, volume, and temperature of particles) and the NPT set (constant number, pressure, and temperature of particles). Finally, the final protein–ligand complex model of MD simulation was obtained by generating trajectories (Lin et al., 2019).

## 3 Results and Discussion

### 3.1 Network Pharmacology Analysis

#### 3.1.1 GO/KEGG Analysis Results

**FIGURE 3:**
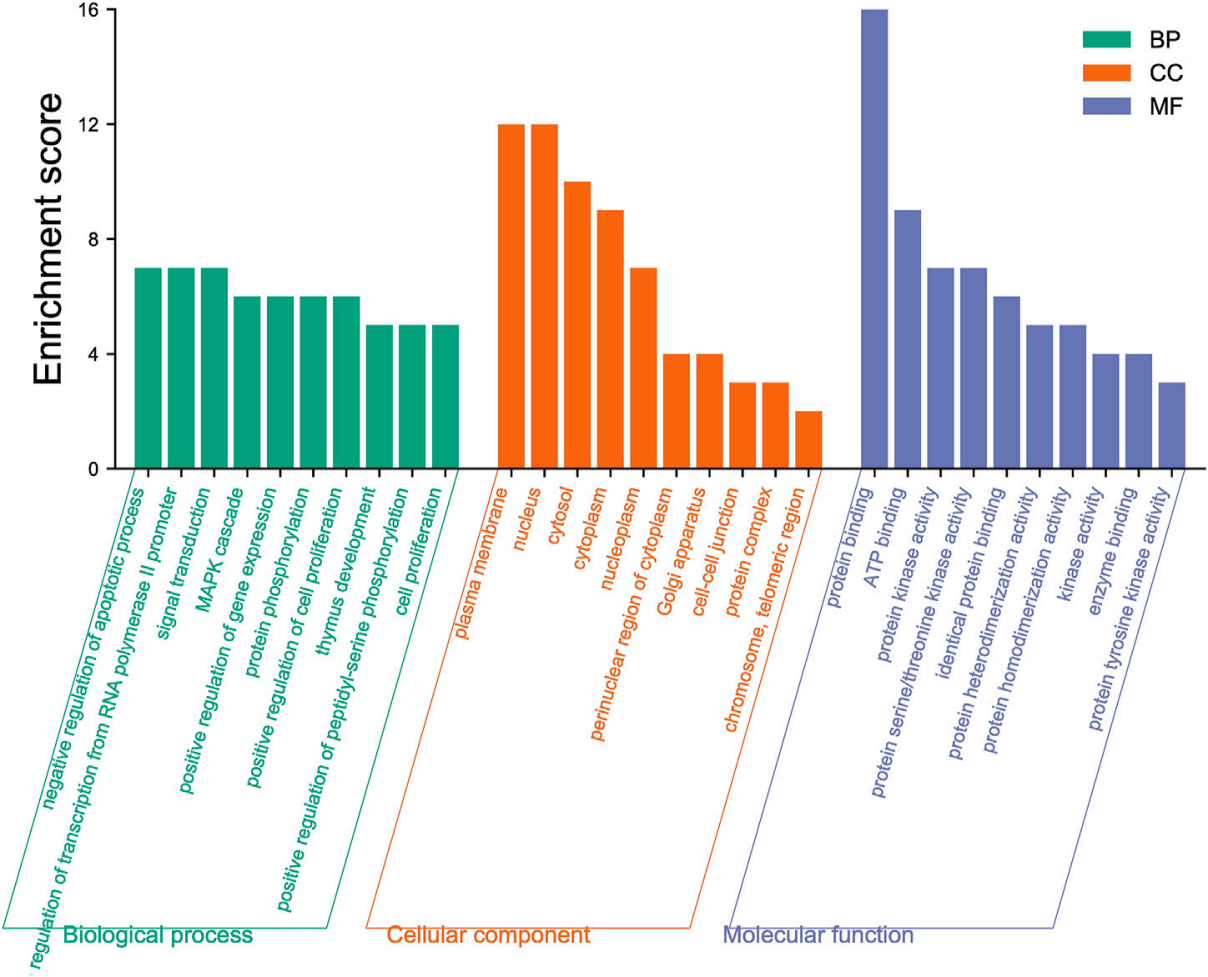
Top ten results of GO functional enrichment of biological processes (BP), cell components (CC), and molecular functions (MF).

The 18 intersection targets were inputted into the DAVID database for GO and KEGG bioenrichment analysis. As shown in Figure 3, GO analysis results showed that the involved biological processes (BP) mainly include the processes of positive regulation of transcription from RNA polymerase II promoter, negative regulation of the apoptotic process, signal transduction, MAPK cascade, positive regulation of gene expression, and protein phosphorylation. The cell components (CC) involved mainly include the plasma membrane, nucleus, cytosol, cytoplasm, nucleoplasm, perinuclear region of the cytoplasm, and other components. The molecular functions (MF) involved mainly included the functions of protein binding, ATP binding, protein kinase activity, protein serine/threonine kinase activity, identical protein binding, and protein heterodimerization activity.

**FIGURE 4:**
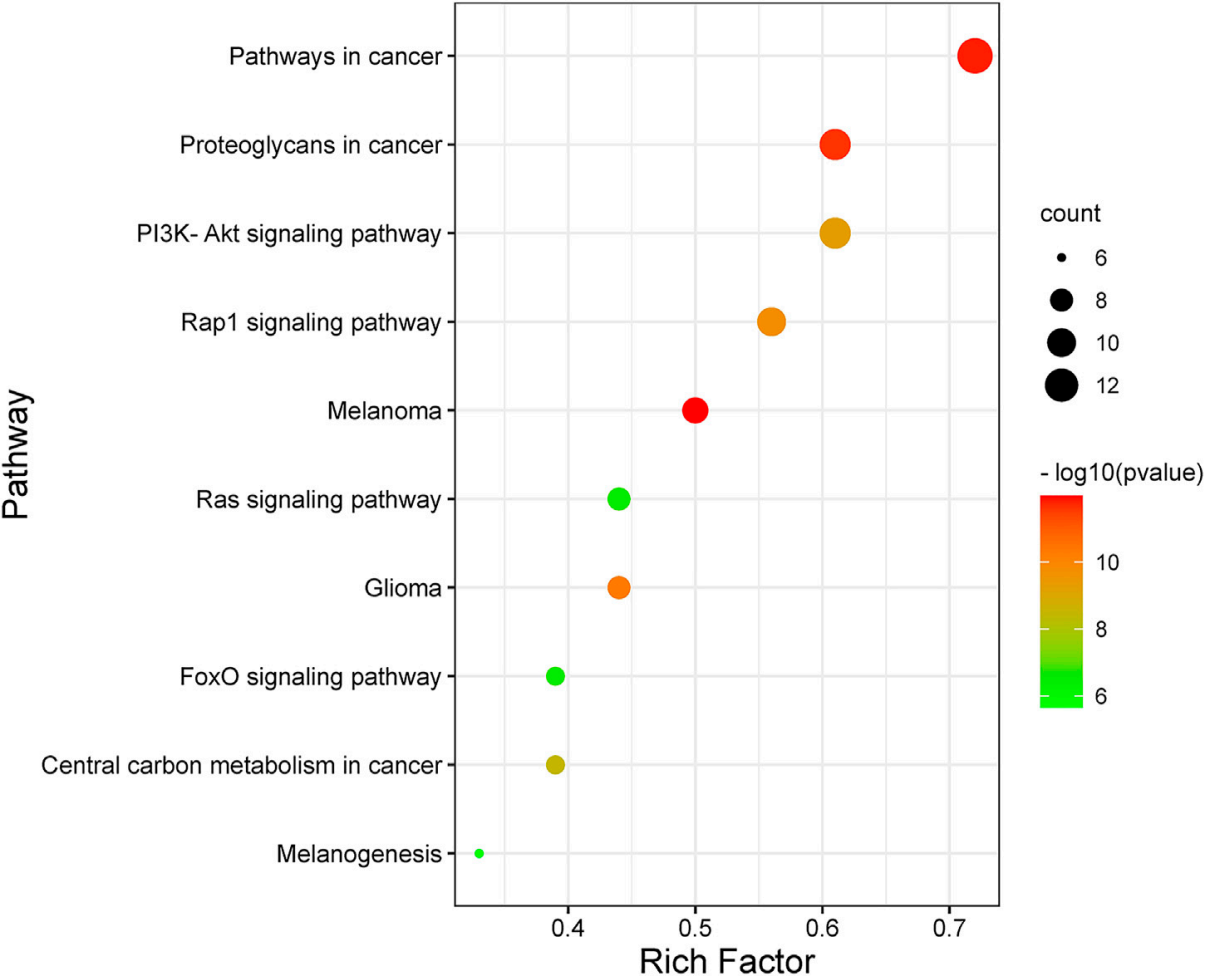
Bubble diagram of KEGG pathway enrichment.

By analyzing the metabolic pathway of KEGG, 72 signaling pathways were enriched, of which 70 pathways were qualified (*p* < 0.05). Figure 4 shows the ten major pathways of LCF anti-melanoma. The main channels involved were pathways in cancer, proteoglycans in cancer, PI3K-Akt signaling pathway, Rap1 signaling pathway, melanoma, glioma, Ras signaling pathway, central carbon metabolism in cancer, Fox O signaling pathway, melanogenesis, and so on. These key pathways can regulate the proliferation and apoptosis of melanoma cells and participate in the development of melanoma. At the same time, we found that Tyr, Map2k1, Kit, Ctnb1, Raf1, Hras, and other key targets regulate several key pathways together to play a therapeutic role.

#### 3.1.2 Compound-Target-Pathway Network Construction

**FIGURE 5:**
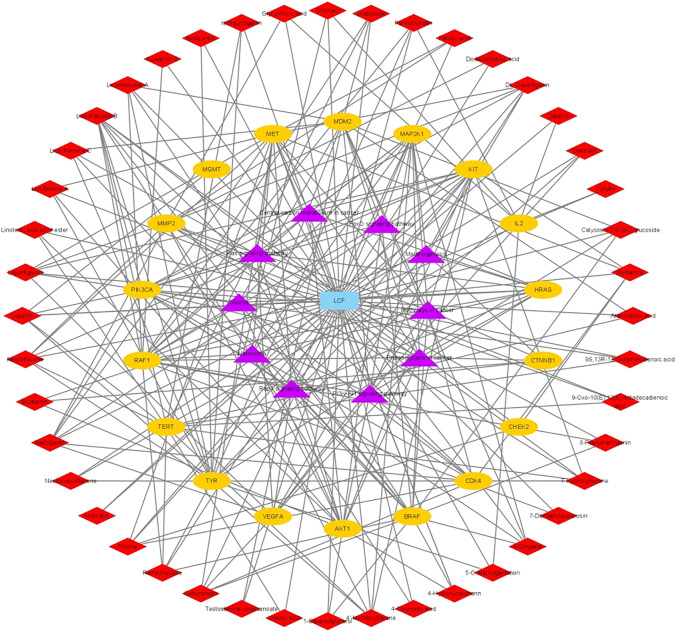
“C-T-P” network.

**FIGURE 6:**
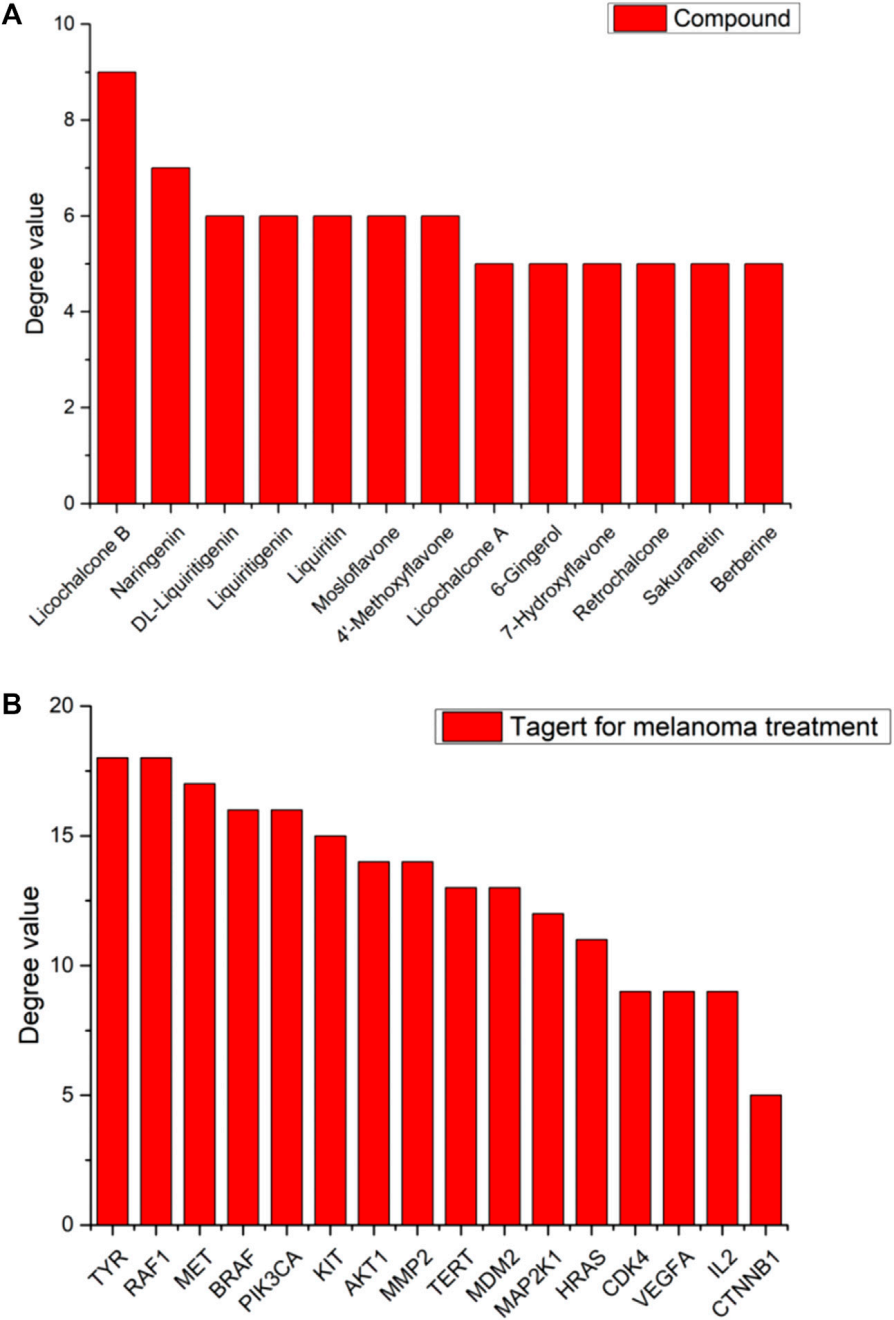
Distribution of degree values of partial compounds **(A)** and target **(B)** in Network Diagram.

To better understand the mechanism of LCF in the treatment of melanoma, we used Cytoscape 3.8.2 to build a “C-T-P” network diagram (Figure 5) and analyzed the degree values of various parts through a histogram (Figure 6; Tables 1, 2). In the network, the triangle represents the signaling pathway, the circle represents the target protein, and the diamond represents the active component. In the histogram, licochalcone B, naringenin, DL-liquiritigenin, liquiritigenin, liquiritin, mosloflavone, 4′-methoxyflavone, licochalcone A, 6-gingerol, 7-hydroxyflavone, retrochalcone, sakuranetin, and berberine showed higher degree values, indicating that these active ingredients play a major role in the process of anti-melanoma (Figure 6A). The potential targets of LCF in the treatment of melanoma show higher values of Tyr, Raf1, and Met (Figure 6B), indicating that these targets were the key targets of melanoma resistance. Therefore, this study suggests that the anti-melanoma mechanism of LCF was the result of multi-compound and multi-target interactions.

**TABLE 1:** Top 20 potentially effective compounds in the prescription.

PubChem ID	Compound	Degree
5318999	Licochalcone B	9
932	Naringenin	7
1889	DL-Liquiritigenin	6
114829	Liquiritigenin	6
503737	Liquiritin	6
471722	Mosloflavone	6
77793	4′-Methoxyflavone	6
5318998	Licochalcone A	5
442793	6-Gingerol	5
5281894	7-Hydroxyflavone	5
6442675	Retrochalcone	5
73571	Sakuranetin	5
2353	Berberine	5
5280378	Formononetin	4
9840805	Licochalcone C	4
5319000	Licoflavone A	4
54682930	4-Hydroxycoumarin	4
90479675	Glabrolide	4
5281708	Daidzein	3
445858	Ferulic acid	3

**TABLE 2:** Potential therapeutic targets.

Gene official symbol	Degree
TYR	18
RAF1	18
MET	17
BRAF	16
PIK3CA	16
KIT	15
AKT1	14
MMP2	14
TERT	13
MDM2	13
MAP2K1	12
HRAS	11
CDK4	9
VEGFA	9
IL2	9
CTNNB1	5
CHEK2	3
MGMT	1

### 3.2 Analysis of the 2D-QSAR Pharmacophore Model

Through the network pharmacological screening, we found that the degree value of the Tyr target was the first, which indicates that it may be the main target of LCF anti-melanoma, and a large number of reports also confirmed this view (Solano et al., 2006; Jawaid et al., 2009; Gao, 2018; Pu et al., 2020). Therefore, this part uses this target corresponding tyrosinase as the activity research.

#### 3.2.1 2D-QSAR Pharmacophore Model With Activity Prediction

The 2D-QSAR constructed by the MLR/PLS method can predict the activity of unknown compounds very well. Supplementary Figures S3, S4 show the linear regression equation of the 2D-QSAR model established by multiple linear regression (MLR) and partial least squares (PLS). The correlation coefficients (r^2^) of the training set compounds constructed by the MLR/PLS method were 0.984 and 0.894, respectively, which showed that the model constructed by the MLR method had better predictive ability than that constructed by the PLS method. Based on the established 2D-QSAR pharmacophore model, the test sets were used for validation, and the experimental and predictive activities (semi-inhibitory concentrations) of these test sets are listed in Table 3. The logIC_50_ of the test set can be divided into three groups: logIC_50_ < 0, high activity; 0 ≤ logIC_50_ ≤ 2.176, medium activity; and logIC_50_ > 2.176, low activity or inactivity (Gao, 2018). It can be seen from Table 3 that the model compounds based on MLR have large quality errors, most compounds have low accuracy, and only a few of them can be accurately predicted. Except for a few compounds, the 2D-QSAR pharmacophore model established by PLS can correctly predict the activities of other compounds. Therefore, compared with the model established by MLR, the model established by PLS shows good accuracy.

**TABLE 3:** Based on the 2D-QSAR test set compound experimental and predicted activity logIC_50_.

Compound no.	Experiment logIC_50_	Predicted (MLRModel) logIC_50_	Predicted (PLSModel) logIC_50_
Tyr-21	−0.77	−0.59	−0.74
Tyr-22	0.77	1.14	1.04
Tyr-23	0.90	0.39	−0.02
Tyr-24	1.21	1.81	0.73
Tyr-25	1.46	1.56	1.57
Tyr-26	1.50	1.69	1.46
Tyr-27	1.56	1.37	1.76
Tyr-28	1.76	2.38	1.36
Tyr-29	1.80	2.01	2.28
Tyr-30	2.05	2.40	2.25
Tyr-31	2.18	2.34	1.97
Tyr-32	2.41	1.50	2.29

#### 3.2.2 Prediction of LCF Activity Based on the 2D-QSAR Pharmacophore Model

In order to explore the structural characteristics of LCF anti-melanoma, this part used the 2D-QSAR pharmacophore model constructed by the MLR/PLS method to predict the anti-melanoma activity of LCF compounds. Table 4 shows the predicted activity of the top 20 LCF compounds screened by network pharmacology (logIC_50_). As shown in Table 4, licochalcone A, 6-gingerol, retrochalcone, formononetin, licoflavone A, and daidzein showed higher activity in the model established by MLR, with logIC_50_ values of −0.37, −0.42, −0.38, −0.38, −0.38, and −0.38, respectively. Similarly, the IC50 values of licochalcone B, licochalcone A, 6-gingerol, retrochalcone, formononetin, licoflavone A, and daidzein in the PLS model were −0.056, −0.14, −0.34, −0.11, −0.11 −0.11, and −0.11 and showed higher activity, respectively. In the prediction of the two models, except for licochalcone B, the predicted activity values of other compounds were basically the same. Therefore, although the 2D-QSAR pharmacophore model established by MLR in the validation of the test set shows the poor predictive effect, it still has a good predictive ability. In addition, since the model established by PLS has better accuracy, we selected the predicted values of the 2D-QSAR pharmacophore model constructed by the PLS method as the follow-up study.

**TABLE 4:** Based on the 2D-QSAR model to predict the activity logIC_50_ of LCF.

PubChem ID	Compound	Predicted (MLRModel) logIC_50_	Predicted (PLSModel) logIC_50_
5318999	Licochalcone B	0.26	−0.06
932	Naringenin	2.36	1.82
1889	DL-Liquiritigenin	1.92	1.83
114829	Liquiritigenin	1.92	1.83
503737	Liquiritin	3.42	1.92
471722	Mosloflavone	3.24	2.44
77793	4′-Methoxyflavone	3.44	2.68
5318998	Licochalcone A	−0.37	−0.13
442793	6-Gingerol	−0.42	−0.34
5281894	7-Hydroxyflavone	2.20	2.06
6442675	Retrochalcone	−0.38	−0.11
73571	Sakuranetin	2.65	1.96
2353	Berberine	3.74	2.89
5280378	Formononetin	−0.38	−0.11
9840805	Licochalcone C	2.65	1.96
5319000	Licoflavone A	−0.38	−0.11
54682930	4-Hydroxycoumarin	2.65	1.96
90479675	Glabrolide	3.74	2.89
5281708	Daidzein	−0.38	−0.11
445858	Ferulic acid	2.65	1.96

In conclusion, seven compounds with high activity were screened from the 2D-QSAR pharmacophore model constructed by the MLR/PLS method in this study (Supplementary Figure S5). Meanwhile, this study showed that LCF has good anti-melanoma activity and provides theoretical guidance for clinical research of LCF anti-melanoma.

### 3.3 3D-QSAR Pharmacophore Model Analysis

Although the 2D-QSAR pharmacophore model has successfully predicted the high activity characteristics of seven compounds, the structure–activity relationship between the common structures of these seven compounds and diseases was not known. Therefore, this part will use the 3D-QSAR pharmacophore model to explore the structure–activity relationship of LCF anti-melanoma.

#### 3.3.1 Construction of the 3D-QSAR Pharmacophore Model by the HipHop Method

In order to elucidate the structure–activity relationship of LCF anti-melanoma and search for the best pharmacophore model of LCF against melanoma, a total of 10 3D-QSAR pharmacophore models were generated using the HipHop method in this part. Table 5 shows the matching degree of 10 pharmacophore models with seven compounds with high activity in LCF. Each row in the table represents a pharmacophore. As shown in the table, the first pharmacophore has the higher score, so “pharmacophore 01” can be selected as the optimal pharmacophore for the follow-up study. Among them, the features in “pharmacophore 01” are HDDA, indicating that this pharmacophore contains one hydrophobic feature, two hydrogen bond donor features, and one hydrogen bond acceptor feature. The spatial arrangement of the pharmacophore is shown in Supplementary Figure S6. The rank indicated a score of 64.452 for this pharmacophore; Direct Hit indicated that the pharmacophore characteristics matched with seven small molecules. Partial Hit indicates that the number of partial matching pharmacophores with seven small molecules is 0. Max Fit indicated that each of the four pharmacophores could be matched with seven small molecules. Therefore, this study showed that “pharmacophore 01” is the best model of the anti-melanoma pharmacophore of LCF.

**TABLE 5:** Parameters of 10 common features of the pharmacophore.

Pharmacophore	Feature	Rank	Direct Hit	Partial Hit	Max Hit
01	HDDA	64.452	1111111	0000000	4
02	HDDA	64.393	1111111	0000000	4
03	HDDA	64.393	1111111	0000000	4
04	HDDA	63.658	1111111	0000000	4
05	HDDA	63.658	1111111	0000000	4
06	HDDA	63.532	1111111	0000000	4
07	HDDA	63.532	1111111	0000000	4
08	HAAA	63.052	1111111	0000000	4
09	HAAA	62.993	1111111	0000000	4
10	HAAA	62.993	1111111	0000000	4

#### 3.3.2 Construction of the 3D-QSAR Model by the Energy Grid Points Method

**FIGURE 7:**
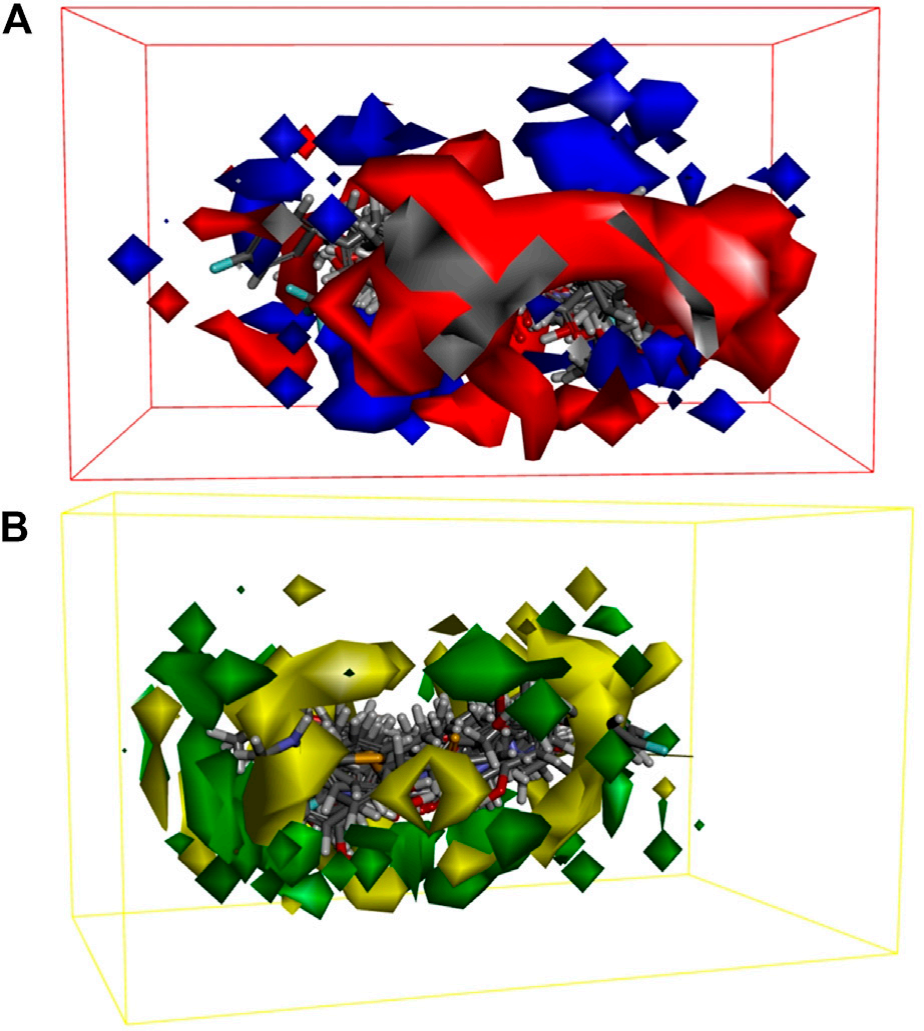
Electrostatic field coefficient contour map **(A)** and the stereo field coefficient contour map **(B)** of the training set molecules matched to the 3D-QSAR model.

In order to further elucidate the structure–activity relationship of LCF anti-melanoma, non-covalent interaction between LCF and anti-melanoma targets was explored. The 3D-QSAR model, which uses energy grid points as a descriptor, is a regression model based on small molecule steric and electrostatic fields and can be used to predict the activity of unknown small ligand molecules and to observe receptor–ligand interactions, both favorable and unfavorable (Potemkin et al., 2017). Figure 7A shows the contour map of the electrostatic field coefficients of the training set molecules matched to the 3D-QSAR model. Among them, the red area indicates that the stronger the negative charge of the substituents in this area, the better the activity of the compound; the blue area indicates that the stronger the positive charge of the substituents in this area, the better the activity of the compound. Figure 7B shows the contour map of the stereo field coefficients of the training set molecules matched to the 3D-QSAR model. Among them, the yellow area indicates that the increase in the volume of the substituents in this area was not conducive to improving the activity of the compound; the blue area indicates that the increase in the volume of the substituents in this area was conducive to improving the activity of the compound. This study showed that both the shape of the compound molecule and its electrostatic distribution have an effect on the activity of the compound. Therefore, we can screen the active compounds in LCF based on this information to find better drug molecules. Meanwhile, this study also showed that LCF has better anti-melanoma activity by producing non-covalent bond interaction with melanoma-related targets.

#### 3.3.3 3D-QSAR Pharmacophore Verification

**FIGURE 8:**
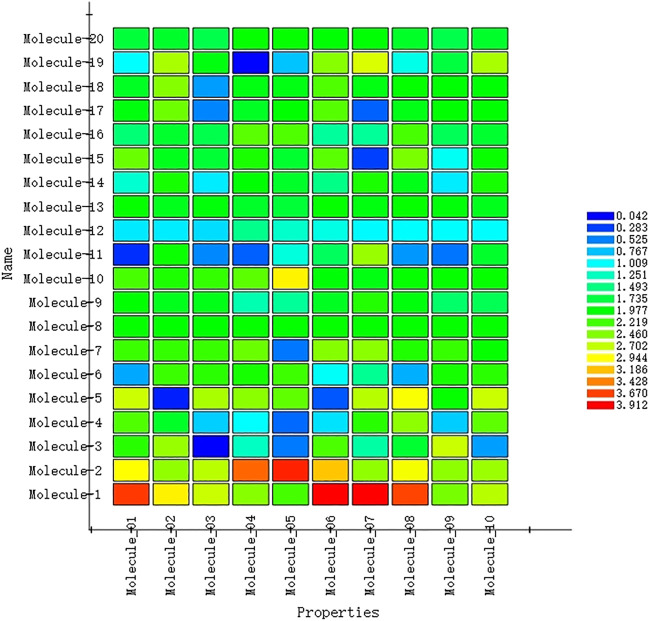
Heat map of the predicted compounds by the 10 pharmacophore models for the test set compounds.

In order to verify the validity of the 3D-QSAR potency model constructed by HipHop, we need to verify whether the pharmacophore has a good ability to distinguish between active and inactive molecules through a known training set of compounds with activity. Figure 8 shows the thermal maps of ten 3D-QSAR pharmacophore models for predicting training set compounds. Table 6 shows the corresponding matching degree. The closer the fit value is to 4, the better the compound fit with the model; the closer the fit value is to 0, the less the compound fit with the model. As shown in Figure 8, the FitValue values of the compounds with higher activity in “pharmacophore 01” were higher than those of the compounds with lower activity in “pharmacophore 01”. This result indicates that “pharmacophore 01” was the best 3D-QSAR pharmacophore model for predicting the activity of LCF compounds. It was also suggested that the pharmacophore model may be the decisive therapeutic pharmacophore of LCF anti-melanoma.

**TABLE 6:** Matching degree of the test set compounds predicted by the 10 pharmacophore models.

Compound no.	01	02	03	04	05	06	07	08	09	10
Tyr-1	3.70	3.00	2.74	2.49	2.24	3.91	3.91	3.66	2.43	2.66
Tyr-2	2.95	2.53	2.68	3.52	3.78	3.17	2.52	2.90	2.51	2.56
Tyr-3	2.12	2.55	0.04	1.24	0.50	2.26	1.35	1.79	2.73	0.64
Tyr-4	2.27	1.83	0.82	1.02	0.43	0.89	2.13	2.51	0.812	2.33
Tyr-5	2.74	0.157	2.61	2.50	2.34	0.36	2.67	2.92	2.00	2.73
Tyr-6	0.68	2.20	2.14	2.06	2.21	1.04	1.42	0.71	2.06	2.14
Tyr-7	2.20	2.18	2.19	2.38	0.49	2.48	2.499	2.10	2.18	2.00
Tyr-8	2.00	1.99	1.99	2.00	1.99	2.00	2.00	2.00	2.00	2.00
Tyr-9	1.98	1.88	1.89	1.32	1.40	1.85	2.11	1.93	1.58	1.66
Tyr-10	2.25	2.08	2.17	2.32	2.99	1.95	1.91	2.00	2.00	2.00
Tyr-11	0.22	2.02	0.56	0.40	1.14	1.63	2.55	0.62	0.48	1.81
Tyr-12	0.92	0.93	0.87	1.45	1.21	1.11	0.98	1.00	1.00	1.00
Tyr-13	2.00	1.87	1.95	1.82	1.77	2.00	1.92	2.00	1.95	1.87
Tyr-14	1.19	2.06	0.93	1.96	1.82	1.47	2.07	1.89	0.93	2.06
Tyr-15	2.38	1.87	1.77	2.08	1.80	2.32	0.28	2.43	1.05	2.01
Tyr-16	1.54	1.81	1.69	2.36	2.27	1.39	1.41	2.24	1.65	1.80
Tyr-17	1.93	2.39	0.54	1.85	1.97	2.29	0.41	1.90	1.98	1.98
Tyr-18	1.84	2.48	0.64	1.92	1.90	2.28	1.91	1.99	1.96	1.96
Tyr-19	1.01	2.62	1.92	0.06	0.78	2.49	2.79	1.10	1.74	2.62
Tyr-20	1.76	1.81	1.70	2.00	2.00	1.97	1.97	1.83	1.70	1.81

In conclusion, logIC_50_ values of the top 20 compounds selected by network pharmacology were predicted by the 2D-QSAR pharmacophore model, and seven highly active compounds were screened successfully. Then, an optimal 3D-QSAR pharmacophore model for predicting the activity of LCF compounds was constructed by the HipHop method. Finally, the effectiveness of the 3D-QSAR pharmacophore was verified by a training set of compounds with known activity, and the possible decisive therapeutic effect of the “pharmacophore 01” was speculated. Therefore, we will carry on with the molecular docking and the MD simulation to carry on with the verification of it, further discussing the LCF anti-melanoma molecular mechanism.

### 3.4 Molecular Docking Verification

**FIGURE 9:**
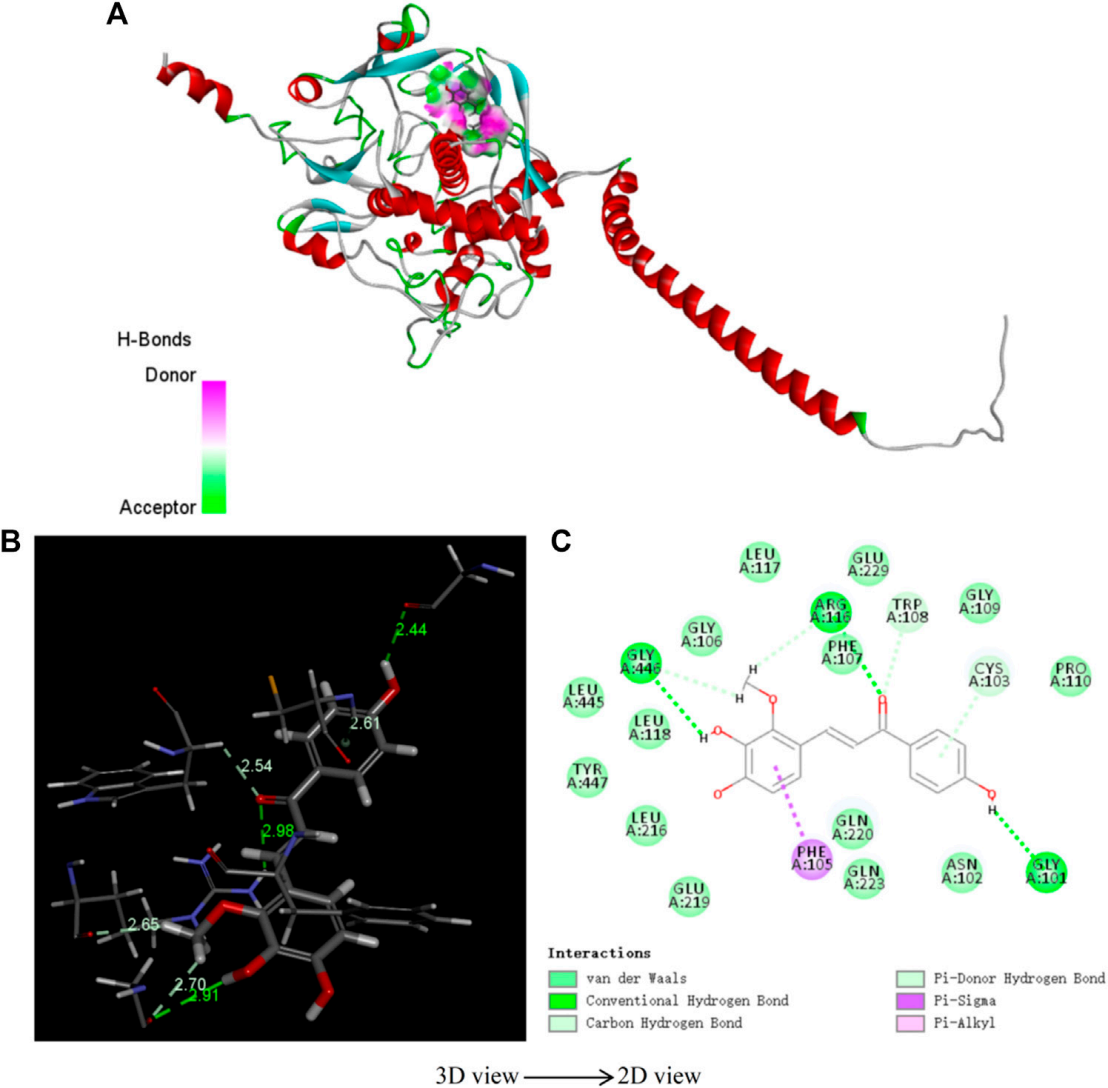
3D structure **(A)**, spatial structure **(B)**, 2D structure **(C)** of tyrosinase and licochalcone B molecule docking.

In order to verify the rationality of the pharmacophore model constructed by seven highly active compounds of LCF, molecular docking was used to verify the model. Table 7 shows the docking scores and docking energies of licochalcone B, licochalcone A, 6-gingerol, retrochalcone, formononetin, licoflavone A, daidzein, and tyrosinase. The results of docking showed that the seven highly active compounds had a good binding activity with tyrosinase. Among them, licochalcone B, licochalcone A, 6-gingerol, retrochalcone, formononetin, and licoflavone A all scored more than 100 docking points, indicating that these six compounds play an important role in the anti-melanoma process of LCF. Figure 9 shows the molecular docking of licochalcone B with tyrosinase. The combination of tyrosinase and licochalcone B occurs in the form of hydrogen bonds and π bonds. The interaction groups were consistent with the “pharmacophore 01”. Specifically, the hydrophobic region of the benzene ring of licochalcone B interacts with the amino acid residue PHE on tyrosinase to form a π bond with a spacing of 2.61Å. The two hydroxyl hydrogen bond donors on licochalcone B interacted with the GLY of the amino acid residues on tyrosinase to form the hydrogen bond spacing of 2.91Å and 2.44Å. The carbonyl hydrogen bond receptors on the licochalcone B compound interact with the amino acid residue PHE on the tyrosinase to form a hydrogen bond spacing of 2.98Å (Figures 9B,C). Similarly, Supplementary Figure S7 shows the results of licochalcone A, 6-gingerol, retrochalcone, formononetin, licoflavone A, daidzein, and tyrosinase in much the same way as licochalcone B. Therefore, molecular docking studies have shown that the optimal “pharmacophore 01” constructed by the 3D-QSAR pharmacophore model is the dominant pharmacophore in the anti-melanoma activity of LCF.

**TABLE 7:** Molecular docking.

Compound	Protein	Libdock score	Binding energy (kcal/mol)	Ligand energy (kcal/mol)	Protein energy (kcal/mol)	Complex energy (kcal/mol)
Licochalcone B	Tyrosinase (EC 1.14.18.1)	107.5	41.24	8294.06	−21533.3	−13198
Licochalcone A	Tyrosinase (EC 1.14.18.1)	121.1	2153.06	432.33	−21533.3	−18947.9
6-Gingerol	Tyrosinase (EC 1.14.18.1)	131.4	1784.29	29.59	−21533.3	−19719.5
Retrochalcone	Tyrosinase (EC 1.14.18.1)	111.9	353.69	76.74	−21533.3	−21102.9
Formononetin	Tyrosinase (EC 1.14.18.1)	116.9	82.20	55.96	−21533.3	-21395.2
Licoflavone A	Tyrosinase (EC 1.14.18.1)	128.4	664.88	55.46	−21533.3	−20813
Daidzein	Tyrosinase (EC 1.14.18.1)	95.8	10924.9	32.94	−21533.3	−10575.5

### 3.5 MD Simulation

**FIGURE 10:**
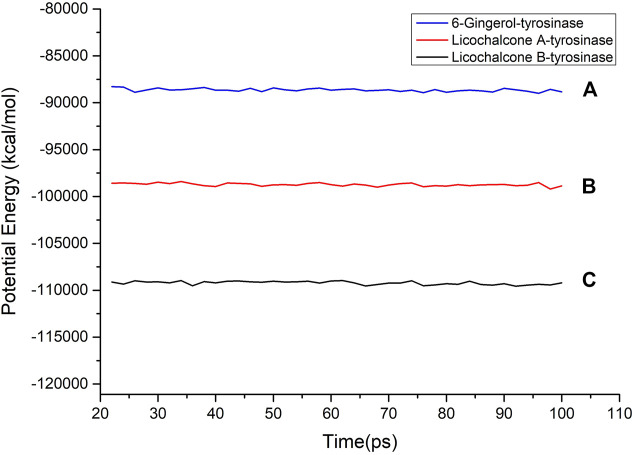
Potential energy of licochalcone B–tyrosinase **(A)**, licochalcone A–tyrosinase **(B)**, and 6-gingerol–tyrosinase **(C)**.

**FIGURE 11:**
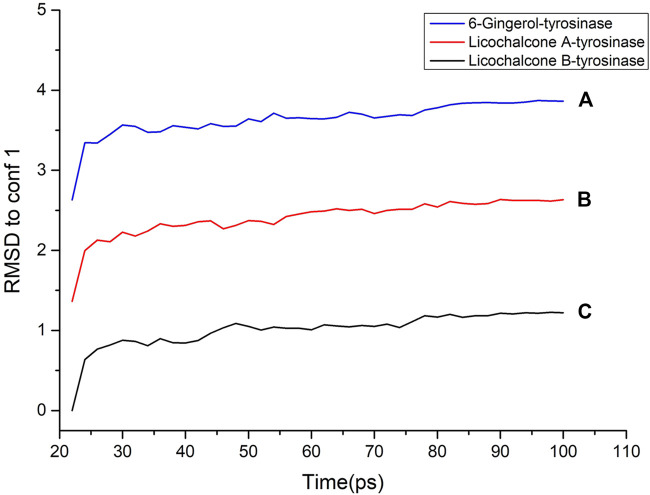
RMSD to conf 1 of licochalcone B–tyrosinase **(A)**, licochalcone A–tyrosinase **(B)**, and 6-gingerol–tyrosinase **(C)**.

**FIGURE 12:**
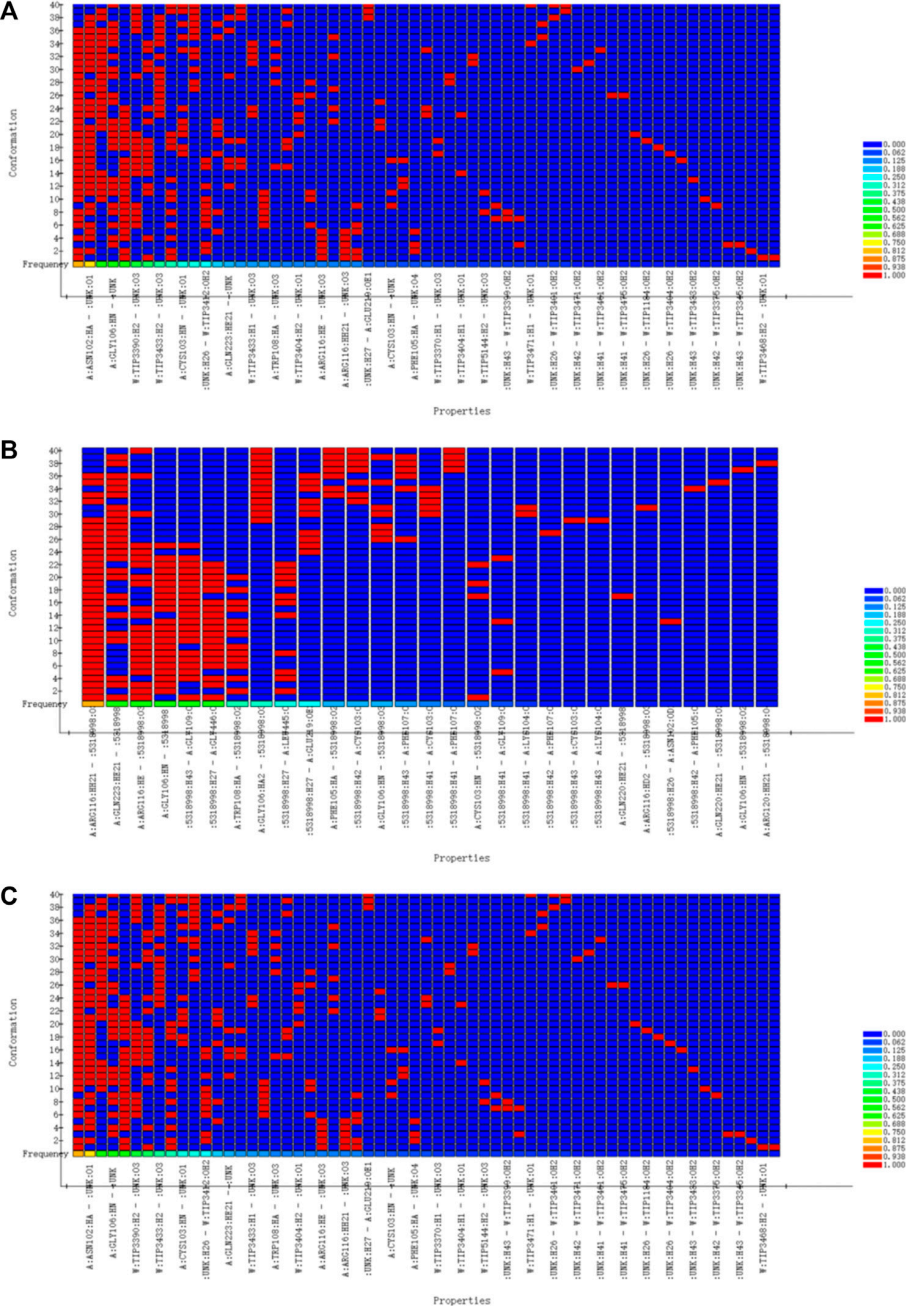
Hydrogen bond heat map of licochalcone B-tyrosinase **(A)**, licochalcone A-tyrosinase **(B)**, 6-gingerol-tyrosinase **(C)**.

Molecular dynamics simulation is a key theoretical method, which can be used to obtain the stability of the protein–ligand complex. In order to determine the binding mass of small molecular ligands with tyrosinase after docking, the complex was further analyzed by molecular dynamics simulation. The RMSD curves, potential energies, and hydrogen bond heat map of licochalcone B, licochalcone A, 6-gingerol, and tyrosinase complexes are shown in Figures 10–12. It can be seen from Figure 10 that after 20 ps, the trajectories of all the complexes tend to balance, and the potential energy tends to stabilize over time. Figure 11 showed that the RMSD curve also exhibits good stability after 80 ps The hydrogen bond heat map also showed that the interaction between ligand compounds and proteins was relatively stable (Figure 12). The MD simulation results showed that hydrogen and π bonds formed between licochalcone B, licochalcone A, 6-gingerol, and tyrosinase help to maintain their stability. The results also prove the rationality of the optimal “pharmacophore 01” constructed by the 3D-QSAR pharmacophore model. Thus, during the treatment phase, licochalcone B, licochalcone A, and 6-gingerol inhibit melanoma formation by interacting with tyrosinase to treat the disease.

## 4 Conclusion

In this study, the molecular mechanism of LCF anti-melanoma was studied by means of network pharmacology, 3D/2D-QSAR, molecular docking, and molecular dynamics (MD) simulation. Network pharmacology studies have shown that licochalcone B, naringenin, and DL-liquiritigenin were key anti-melanoma active components in LCF, and Tyr was the key target of anti-melanoma. 2D-QSAR pharmacophore model studies have shown that licochalcone B, licochalcone A, 6-gingerol, retrochalcone, formononetin, licoflavone A, and daidzein were highly active compounds in the anti-melanoma activity of LCF. The results of the 3D-QSAR model showed that the optimal pharmacophore of LCF was composed of one hydrophobic group, two hydrogen-bonded donor groups, and one hydrogen-bonded acceptor group. Molecular docking studies have shown that the optimal pharmacophore model constructed by 3D-QSAR was the dominant pharmacophore in the anti-melanoma activity of LCF. MD simulations showed that the hydrogen and π bond interactions between licochalcone B, licochalcone A, 6-gingerol, and tyrosinase were helpful to maintain their stability, which proves the rationality of the 3D-QSAR pharmacophore model. In conclusion, this study found the structure–activity relationship between the structural properties and biological activities of LCF, and a reliable statistical model was established to confirm the anti-melanoma activity of LCF.

## Data Availability

The original contributions presented in the study are included in the article/Supplementary Material, further inquiries can be directed to the corresponding authors.
