# Supplementary material for: Investigative on the Molecular Mechanism of Licorice Flavonoids Anti-Melanoma by Network Pharmacology, 3D/2D-QSAR, Molecular Docking, and Molecular Dynamics Simulation
Source: Front Chem. 2022 Mar 2;10:843970. doi: 10.3389/fchem.2022.843970 (PMC8924370; doi:10.3389/fchem.2022.843970)
Supplement: Supplementary file 1 [file DataSheet1.docx]

**[Supplementary information](http://www.rsc.org/suppdata/d0/ra/d0ra06961b/d0ra06961b1.pdf)**

**Investigative on the molecular mechanism of licorice flavonoids anti-melanoma by network pharmacology, 3D/2D-QSAR, molecular docking, molecular dynamics** **simulation**

*Yi Hu^#a^, Yufan Wu^#a^, CuiPing Jiang^a^, Zhuxian Wang^a^, Chunyan Shen^a^, Zhaoming Zhu^a^, Hui Li^b^ Quanfu Zeng^a^,* Yaqi Xue*^a^, Yuan Wang^a^,* *Li Liu**^a^, Yankui Yi**^a^, Hongxia Zhu*^c^, Qiang Liu*^a^*

*^a^School of Traditional Chinese Medicine, Southern Medical University, Guangzhou*

*510515, PR China*

*^b^Department of Traditional Chinese Medicine, Guangzhou Red Cross Hospital, Jinan*

*University, Guangzhou,510220, China*

*^c^**Integrated Hospital of Traditional Chinese Medicine, Southern Medical University,*

*Guangzhou 510315, PR China*

*^#^Yi Hu and Yufan Wu contributed equally to this work.*

**^*^Corresponding author:** School of Traditional Chinese Medicine, Southern Medical University, Guangzhou, 510515, China. Tel.: +86-20-6164-8264, (Qiang Liu)*Email-address*: liuqiang@smu.edu.cn

**^*^Corresponding author:** Integrated Hospital of Traditional Chinese Medicine, Southern Medical University, Guangzhou, 510315, China. Tel.: +86-20-62789408, (Hongxia Zhu)*Email-address*: gzzhx2012@163.com

Table of Contents

**1.[The Tables of main chemical constituents of licorice flavonoids S3](#_bookmark0)**

**2.The tables of Pubchem ID of 74 compounds S7**

**3.The tables of potential therapeutic targets S10**

**4.The figure of venn diagram of coincidence targets S11**

**5.The figure of PPI network S12**

**6.The figure of correlation of MLR on training set compounds S13**

**7. The figure of correlation of PLS on training set compounds S14**

**8.The figure of structural formulae of 7 compounds S15**

**9.The figure of The “pharmacophore 01” with the highest score S16**

**10.Molecular docking S17**

**1.The Tables of main chemical constituents of licorice flavonoids**

Tables. S1 Main chemical constituents in licorice flavonoids

| **NO.** | **Compounds Name** | **Formula** | **Molecular Weight** | **RT**  **（min）** | **Relative contents(%)** |
| --- | --- | --- | --- | --- | --- |
| **1** | 4',7-Dihydroxyflavanone | C15H12O4 | 256.07393 | 3.337 | 1.03 |
| **2** | Licochalcone A | C21H22O4 | 338.15218 | 7.601 | 12.89 |
| **3** | Glycitein | C16H12O5 | 284.06892 | 4.851 | 0.32 |
| **4** | 18-β-Glycyrrhetinic acid | C30H46O4 | 470.34021 | 10.544 | 1.44 |
| **5** | Neobavaisoflavone | C20H18O4 | 322.12098 | 6.619 | 13.78 |
| **6** | Daidzein | C15H10O4 | 254.05818 | 4.447 | 1.71 |
| **7** | Retrochalcone | C16H14O4 | 270.08937 | 7.684 | 0.14 |
| **8** | Isoliquiritin | C21H22O9 | 418.12703 | 4.176 | 0.34 |
| **9** | Glycyrrhizic acid | C42H62O16 | 822.40486 | 6.517 | 0.83 |
| **10** | Diammonium glycyrrhizinate | C42H62O16 | 822.40486 | 6.115 | 5.52 |
| **11** | Osajin | C25H24O5 | 404.16249 | 12.831 | 0.11 |
| **12** | 4'-Methoxyflavone | C16H12O3 | 252.07906 | 7.602 | 0.42 |
| **13** | Liguiritigenin-7-O-β-D-  apiosyl-4'-O-β-D-glucoside | C26H30O13 | 550.16987 | 3.482 | 4.34 |
| **14** | Glabrolide | C30H44O4 | 468.32462 | 11.367 | 0.53 |
| **15** | 7-Hydroxyflavone | C15H10O3 | 238.06307 | 5.707 | 0.02 |
| **16** | Licoflavone A | C20H18O4 | 322.12141 | 6.671 | 4.34 |
| **17** | Liquiritin | C21H22O9 | 418.12727 | 0.822 | 0.16 |
| **18** | Dipotassium glycyrrhizinate | C42H60O16 | 822.40507 | 6.522 | 0.40 |
| **19** | Liquiritigenin | C15H12O4 | 256.074 | 4.737 | 0.67 |
| **20** | Isoliquiritigenin | C15H12O4 | 256.074 | 5.838 | 1.93 |
| **21** | Glabrone | C20H16O5 | 336.1 | 6.359 | 0.13 |
| **22** | Hispaglabridin B | C25H26O4 | 390.1839 | 12.103 | 0.04 |
| **23** | Licochalcone C | C21H22O4 | 338.1524 | 7.746 | 20.44 |
| **24** | Calycosin-7-O-β-D-glucoside | C22H22O10 | 446.1221 | 5.205 | 0.03 |
| **25** | 5-Hydroxy-6,7-dimethoxylflavone | C17H14O5 | 298.0841 | 5.394 | 0.06 |
| **26** | Licochalcone B | C16H14O5 | 286.0843 | 5.363 | 0.04 |
| **27** | Mulberrin | C25H26O6 | 422.1737 | 10.924 | 1.54 |
| **28** | Kuwanon G | C40H36O11 | 692.2271 | 9.199 | 0.01 |
| **29** | Formononetin | C16H12O4 | 268.07393 | 5.955 | 3.50 |
| **30** | Linolenic acid ethyl ester | C20H34O2 | 306.25597 | 12.899 | 0.03 |
| **31** | α-Eleostearic acid | C18H30O2 | 278.22483 | 9.908 | 0.03 |
| **32** | Berberine | C20H17NO4 | 335.11598 | 4.312 | 0.03 |
| **33** | Proline | C5H9NO2 | 115.06339 | 0.891 | 0.01 |
| **34** | α,α-Trehalose | C12H22O11 | 342.11665 | 0.801 | 0.11 |
| **35** | Naringenin | C15H12O5 | 272.06859 | 4.007 | 0.43 |
| **36** | Azelaic acid | C9H16O4 | 188.10472 | 4.228 | 0.02 |
| **37** | 4-Coumaric acid | C9H8O3 | 164.04741 | 3.396 | 0.004 |
| **38** | Ferulic acid | C10H10O4 | 194.05789 | 3.655 | 0.0023 |
| **39** | 5-O-Methylgenistein | C16H12O5 | 284.06871 | 6.953 | 0.07 |
| **40** | Sakuranetin | C16H14O5 | 286.0844 | 4.564 | 0.13 |
| **41** | Testosterone undecanoate | C30H48O3 | 456.36089 | 11.277 | 0.04 |
| **42** | 3-Hydroxybenzoic acid | C7H6O3 | 138.03155 | 2.357 | 0.004 |
| **43** | Daidzin | C21H20O9 | 416.11086 | 3.17 | 0.06 |
| **44** | 4-Hydroxycoumarin | C9H6O3 | 162.03161 | 2.969 | 0.004 |
| **45** | Psoralidin | C20H16O5 | 336.10045 | 8.129 | 0.55 |
| **46** | Nobiletin | C21H22O8 | 402.13191 | 6.684 | 0.03 |
| **47** | 4-O-Methylpinosylvic acid | C16H14O4 | 270.08937 | 6.558 | 0.15 |
| **48** | Anabasine | C10H14N2 | 162.11571 | 1.092 | 0.10 |
| **49** | 6-Acetylcodeine | C20H23NO4 | 341.16304 | 2.299 | 0.01 |
| **50** | Dodecanedioic acid | C12H22O4 | 230.15173 | 6.087 | 0.02 |
| **51** | Ursolic acid | C30H48O3 | 456.36145 | 12.728 | 1.38 |
| **52** | 9-Oxo-10(E),12(E)-  octadecadienoic acid | C18H30O3 | 294.21951 | 10.696 | 0.41 |
| **53** | Palmitoyl ethanolamide | C18H37NO2 | 299.28276 | 12.558 | 0.03 |
| **54** | Choline | C5H13NO | 103.09986 | 0.732 | 0.07 |
| **55** | Oleoyl ethanolamide | C20H39NO2 | 325.29823 | 12.996 | 0.06 |
| **56** | Stearamide | C18H37NO | 283.28761 | 14.543 | 0.34 |
| **57** | 8-Prenylnaringenin | C20H20O5 | 340.13133 | 8.401 | 0.43 |
| **58** | Kanzonol C | C25H28O4 | 392.19914 | 11.574 | 2.09 |
| **59** | 1-Stearoylglycerol | C21H42O4 | 358.30893 | 14.704 | 0.03 |
| **60** | 9S,13R-12-Oxophytodienoic  acid | C18H28O3 | 292.20407 | 7.212 | 0.08 |
| **61** | 7-Hydroxy-3-[4-hydroxy-3-(  3-methyl-2-buten-1-yl)phenyl]-8-(3-methyl-2-buten-1-yl)-4H- chromen-4-one | C25H26O4 | 390.18322 | 8.977 | 2.32 |
| **62** | N,N'-Diphenylguanidine | C13H13N3 | 194.08432 | 0.864 | 0.52 |
| **63** | Arachidonic acid | C20H32O2 | 322.25084 | 12.119 | 0.02 |
| **64** | 7-Demethylsuberosin | C14H14O3 | 230.09426 | 6.175 | 0.24 |
| **65** | 16-Hydroxyhexadecanoic acid | C16H32O3 | 272.23561 | 13.754 | 0.07 |
| **66** | 7-Hydroxy-2-(4- hydroxyphenyl)-4-oxo-3,4- dihydro-2H-chromen-5-yl β-D-  glucopyranoside | C21H22O10 | 434.12183 | 4.342 | 0.01 |
| **67** | Pentadecanoic acid | C15H30O2 | 242.22464 | 13.812 | 0.002 |
| **68** | Lupenone | C30H48O | 424.37087 | 13.118 | 0.009 |
| **69** | 2,2'-Methylenebis(4-methyl-6-  tert-butylphenol) | C23H32O2 | 340.24098 | 13.51 | 0.50 |
| **70** | Methyl hexadecanoate | C17H34O2 | 316.26152 | 8.852 | 0.03 |
| **71** | α-Phenylpiperidine-2-  acetamide | C13H18N2O | 218.14188 | 1.767 | 0.01 |
| **72** | Ritalinic acid | C13H17NO2 | 219.12607 | 1.813 | 0.01 |
| **73** | Corylin | C20H16O4 | 320.10512 | 7.767 | 0.11 |
| **74** | 6-Gingerol | C17H26O4 | 294.18333 | 7.267 | 0.04 |

**2.The tables of Pubchem ID of 74 compounds**

**Table S2** Pubchem ID of 74 compounds

| **NO.** | **Compounds Name** | **Pubchem ID** | **NO.** | **Compounds Name** | **Pubchem ID** |
| --- | --- | --- | --- | --- | --- |
| **1** | 4',7-Dihydroxyflavanone | \| 1889 1889 \| \| --- \| | **2** | Licochalcone A | 5318998 |
| **3** | Glycitein | 5317750 | **4** | 18-β-Glycyrrhetinic acid | [3230](https://pubchem.ncbi.nlm.nih.gov/compound/3230) |
| **5** | Neobavaisoflavone | 5320053 | **6** | Daidzein | 5281708 |
| **7** | Retrochalcone | 6442675 | **8** | Isoliquiritin | 5318591 |
| **9** | Glycyrrhizic acid | 14982 | **10** | Diammonium glycyrrhizinate | 656656 |
| **11** | Osajin | 95168 | **12** | 4'-Methoxyflavone | 77793 |
| **13** | Liguiritigenin-7-O-β-D-  apiosyl-4'-O-β-D-glucoside | 348881191 | **14** | Glabrolide | 90479675 |
| **15** | 7-Hydroxyflavone | 5281894 | **16** | Licoflavone A | 5319000 |
| **17** | Liquiritin | 503737 | **18** | Dipotassium glycyrrhizinate | 656852 |
| **19** | Liquiritigenin | 114829 | **20** | Isoliquiritigenin | 638278 |
| **21** | Glabrone | 5317652 | **22** | Hispaglabridin B | 15228661 |
| **23** | Licochalcone C | 9840805 | **24** | Calycosin-7-O-β-D-glucoside | 9840805 |
| **25** | Mosloflavone | 471722 | **26** | Licochalcone B | 5318999 |
| **27** | Mulberrin | 5481958 | **28** | Kuwanon G | 5281667 |
| **29** | Formononetin | 5280378 | **30** | Linolenic acid ethyl ester | 5367460 |
| **31** | α-Eleostearic acid | 7850014 | **32** | Berberine | 2353 |
| **33** | Proline | 2353 | **34** | α,α-Trehalose | 3231 |
| **35** | Naringenin | 932 | **36** | Azelaic acid | 2266 |
| **37** | 4-Coumaric acid | 637542 | **38** | Ferulic acid | [445858](https://pubchem.ncbi.nlm.nih.gov/compound/445858) |
| **39** | 5-O-Methylgenistein | 5748551 | **40** | Sakuranetin | 73571 |
| **41** | Testosterone undecanoate | 65157 | **42** | 3-Hydroxybenzoic acid | [7420](https://pubchem.ncbi.nlm.nih.gov/compound/7420) |
| **43** | Daidzin | 107971 | **44** | 4-Hydroxycoumarin | 54682930 |
| **45** | Psoralidin | 5281806 | **46** | Nobiletin | 72344 |
| **47** | 4-O-Methylpinosylvic acid | [67322417](https://pubchem.ncbi.nlm.nih.gov/compound/67322417) | **48** | Anabasine | [2181](https://pubchem.ncbi.nlm.nih.gov/compound/2181) |
| **49** | Acetylcodeine | 5486550 | **50** | Dodecanedioic acid | 12736 |
| **51** | Ursolic acid | [64945](https://pubchem.ncbi.nlm.nih.gov/compound/64945) | **52** | 9-Oxo-10(E),12(E)-  octadecadienoic acid | 5283011 |
| **53** | Palmitoyl ethanolamide | 4671 | **54** | Choline | 305 |
| **55** | Oleoyl ethanolamide | 5283454 | **56** | Stearamide | 31292 |
| **57** | 8-Prenylnaringenin | 480764 | **58** | Kanzonol C | 5316802 |
| **59** | 1-Stearoylglycerol | 22035687 | **60** | 9S,13R-12-Oxophytodienoic acid | 14037063 |
| **61** | Erysubin F | 12051847 | **62** | N,N'-Diphenylguanidine | 14657 |
| **63** | Arachidonic acid | 444899 | **64** | 7-Demethylsuberosin | 5316525 |
| **65** | 16-Hydroxyhexadecanoic acid | 10466 | **66** | 7-Hydroxy-2-(4- hydroxyphenyl)-4-oxo-3,4- dihydro-2H-chromen-5-yl β-D-  glucopyranoside | 387123206 |
| **67** | Pentadecanoic acid | 13849 | **68** | Lupenone | 92158 |
| **69** | 2,2'-Methylenebis(4-methyl-6-  tert-butylphenol) | 8398 | **70** | Methyl hexadecanoate | 8181 |
| **71** | α-Phenylpiperidine-2-  acetamide | 386330703 | **72** | Ritalinic acid | 86863 |
| **73** | Corylin | 5316097 | **74** | 6-Gingerol | 442793 |

**3. The tables of potential therapeutic targets**

**Table S3** Potential therapeutic targets

| **Gene Official Symbol** | | | | | | | | |
| --- | --- | --- | --- | --- | --- | --- | --- | --- |
| TYR | MET | MDM2 | PIK3CA | IL2 | CHEK2 | TERT | RAF1 | KIT |
| MMP2 | AKT1 | CTNNB1 | MAP2K1 | CDK4 | HRAS | VEGFA | MGMT | BRAF |

**4. The figure of venn diagram of coincidence targets**


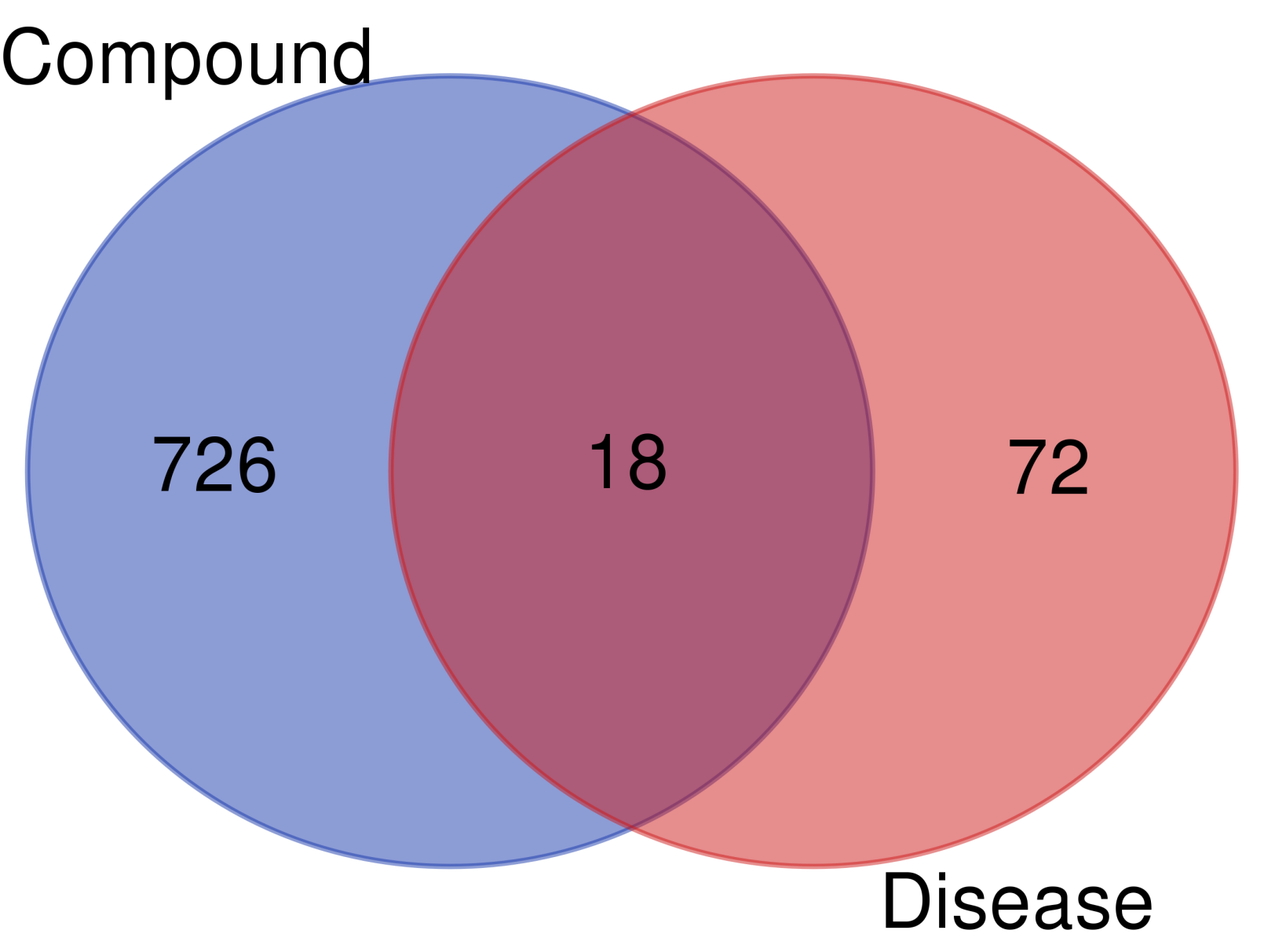


**Figure S1** Venn diagram of coincidence targets

**5. The figure of PPI network**


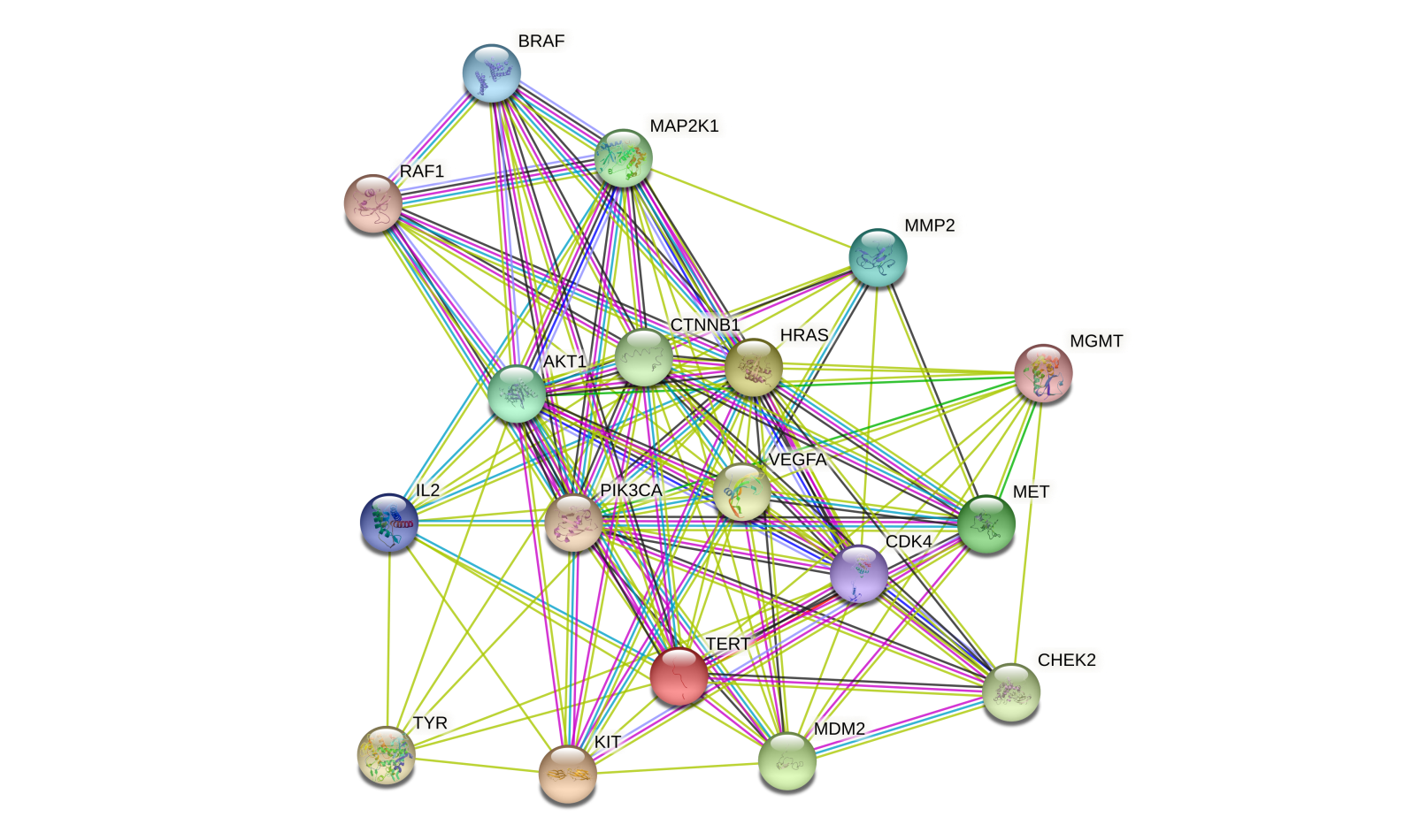


**Figure S2** PPI network

**6. The figure of correlation of MLR on training set compounds**


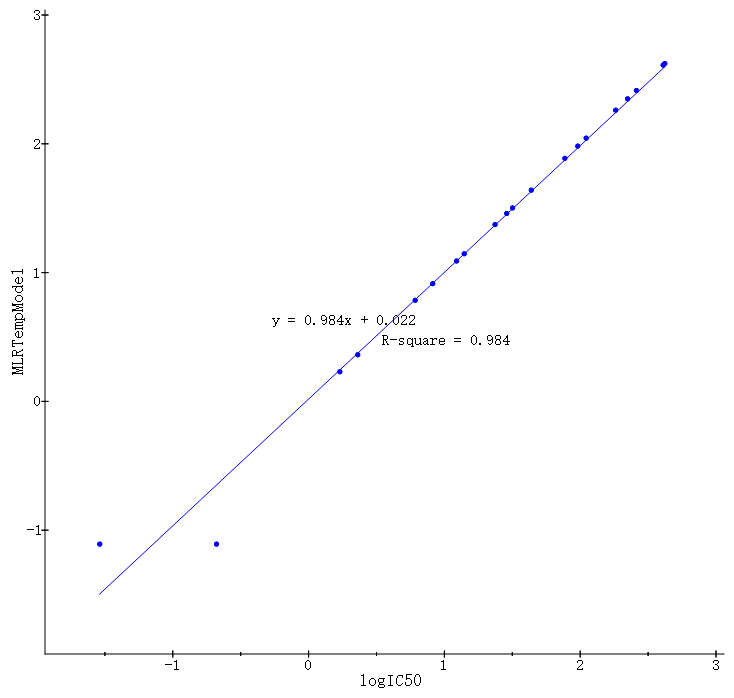


**Figure S3** Correlation between experimental activity and predicted activity (logIC_50_) of MLR on training set compounds.

**7. The figure of correlation of PLS on training set compounds**


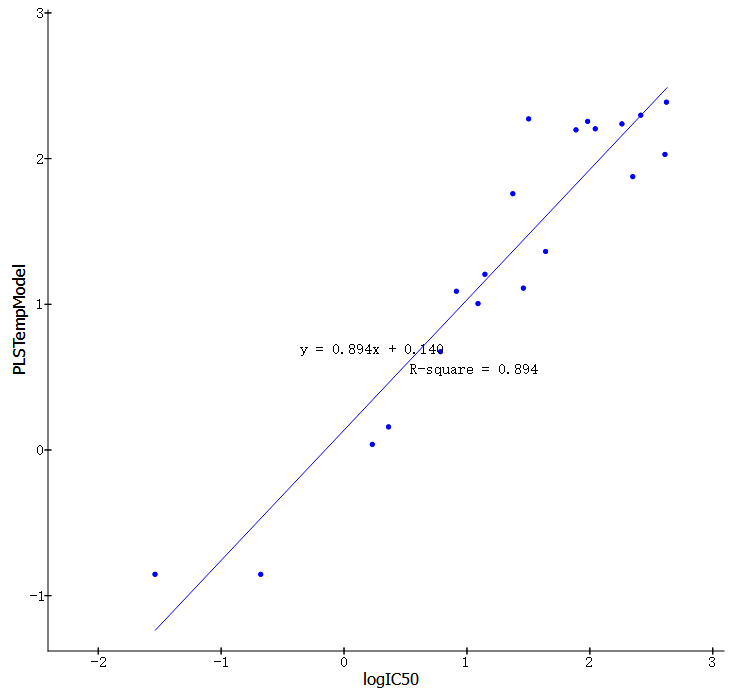


**Figure S4** Correlation between experimental activity and predicted activity (logIC_50_) of PLS on training set compounds.

**8. The figure of structural formulae of 7 compounds**

**
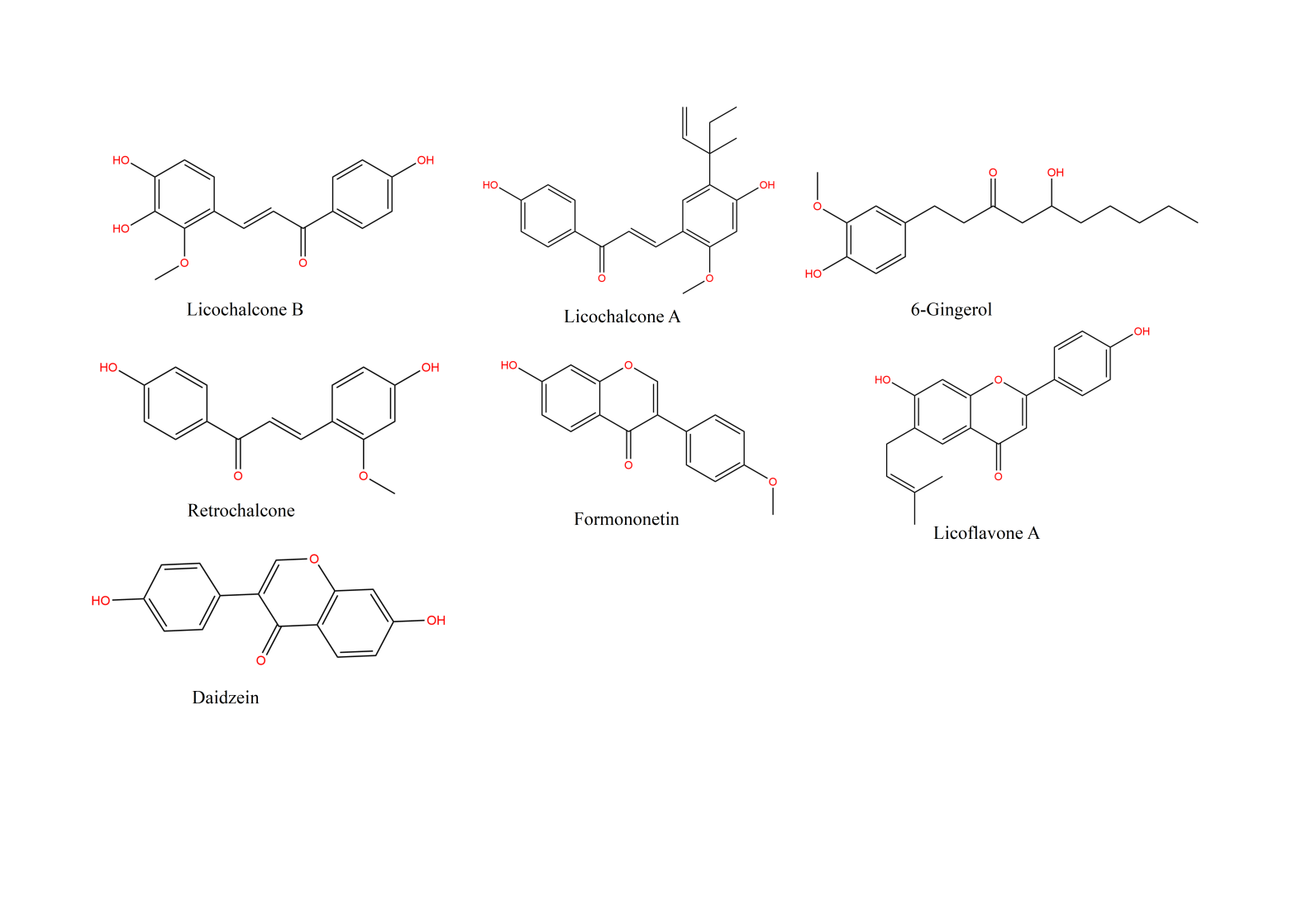
**

**Figure S5** Structural formulae of 7 compounds with high activity in LCF.

**9. The figure of The “pharmacophore 01” with the highest score**
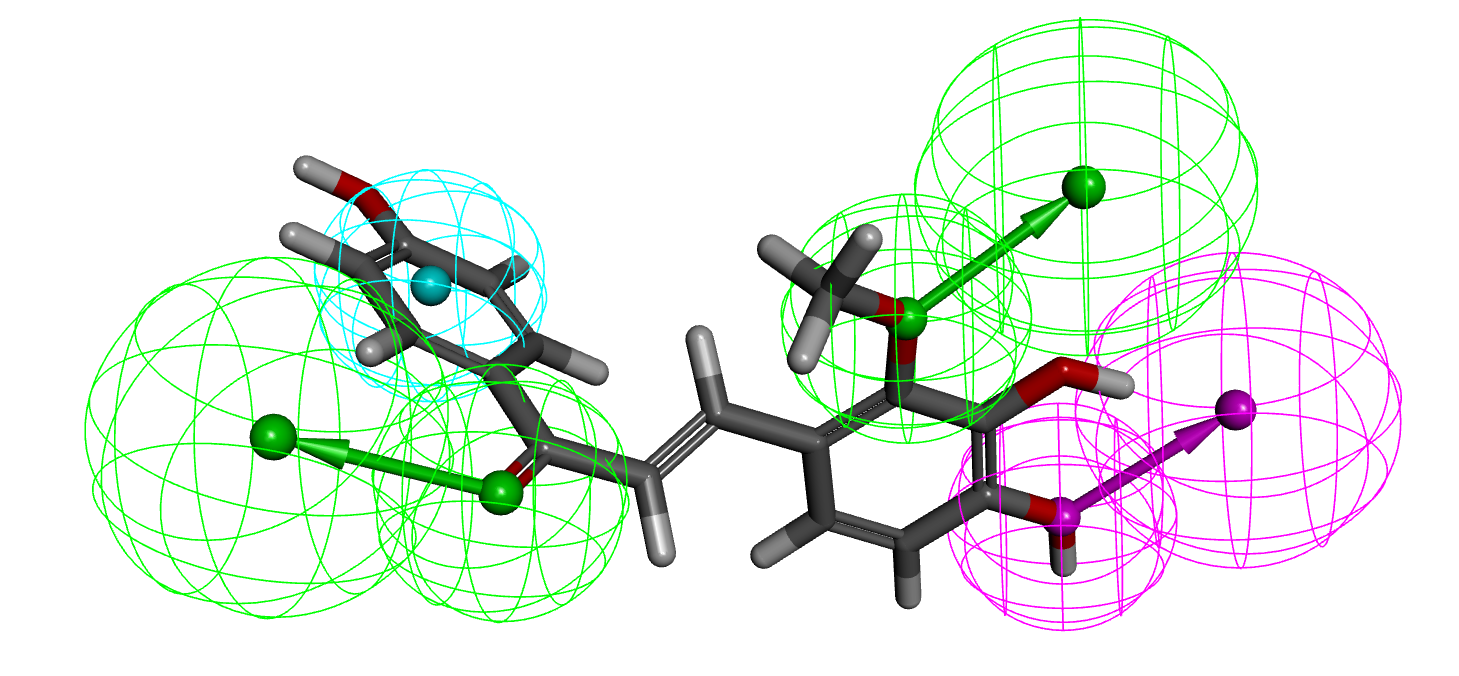


**Figure S6** The “pharmacophore 01” with the highest score is mapped to Licochalcone B (red, hydrogen bond acceptor; green, hydrogen bond donor; blue, hydrophobic).

**10.Molecular docking**


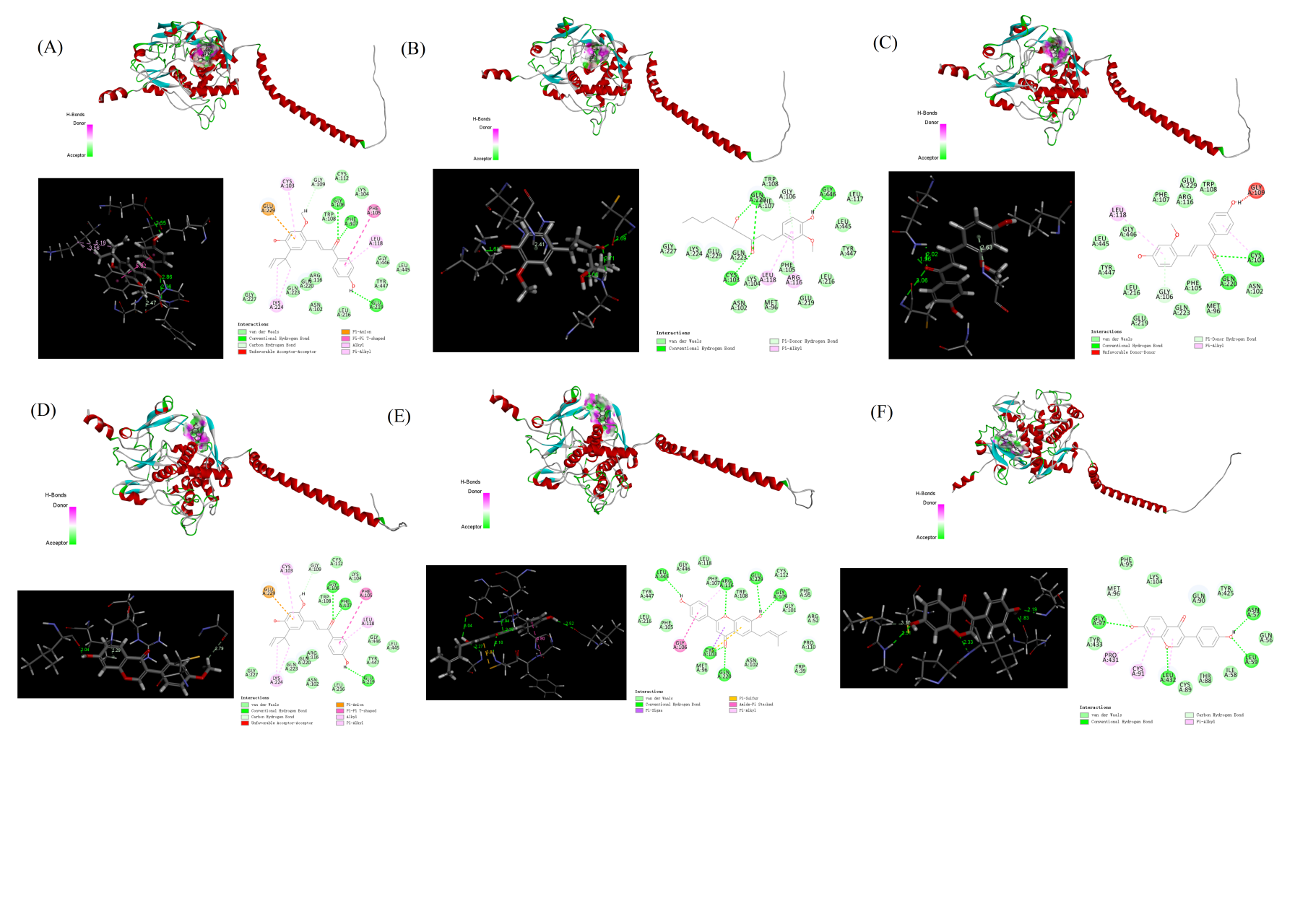


**Figure S7** Molecular docking of Tyrosinase and Licochalcone A (A), 6-Gingerol (B), Retrochalcone (C), Formononetin (D), Licoflavone A (E), Daidzein (F).
